# Targeted analysis of polymorphic loci from low-coverage shotgun sequence data allows accurate genotyping of HLA genes in historical human populations

**DOI:** 10.1038/s41598-020-64312-w

**Published:** 2020-04-30

**Authors:** Federica Pierini, Marcel Nutsua, Lisa Böhme, Onur Özer, Joanna Bonczarowska, Julian Susat, Andre Franke, Almut Nebel, Ben Krause-Kyora, Tobias L. Lenz

**Affiliations:** 1 Research Group for Evolutionary Immunogenomics, Max Planck Institute for Evolutionary Biology, 24306 Ploen, Germany; 2 Institute of Clinical Molecular Biology, Kiel University, 24105 Kiel, Germany; 3 Present Address: Université Paris-Saclay, CNRS, Inria, Laboratoire de recherche en informatique, 91405 Orsay, France

**Keywords:** Evolutionary biology, Genetic markers, Haplotypes, Immunogenetics, Sequencing, Evolutionary genetics, Palaeontology, Population genetics, Adaptive immunity

## Abstract

The highly polymorphic human leukocyte antigen (HLA) plays a crucial role in adaptive immunity and is associated with various complex diseases. Accurate analysis of HLA genes using ancient DNA (aDNA) data is crucial for understanding their role in human adaptation to pathogens. Here, we describe the TARGT pipeline for targeted analysis of polymorphic loci from low-coverage shotgun sequence data. The pipeline was successfully applied to medieval aDNA samples and validated using both simulated aDNA and modern empirical sequence data from the 1000 Genomes Project. Thus the TARGT pipeline enables accurate analysis of HLA polymorphisms in historical (and modern) human populations.

## Introduction

The classical human leukocyte antigen (HLA) genes play a central role in adaptive immunity. They encode for glycoproteins that present antigenic peptides on the cell surface for recognition by immune effector cells, thus enabling the immune system to distinguish between ‘self’ and ‘non-self’, eventually stimulating a specific immune response^1^. Owing to their implication in hundreds of different complex diseases^2,3^, but also because of their importance in human evolution^4,5^, HLA molecules have been extensively studied over the past decades.

HLA genes are among the most polymorphic loci known in the human genome^1,2^. At the molecular level, HLA genetic diversity is characterized by a remarkable amino acid sequence diversification^6,7^ as well as an enhanced rate of non-synonymous substitutions^8^ in the antigen binding groove of HLA molecules (i.e. the pocket where antigens are bound). The specific polymorphism patterns in the exons coding for the antigen binding groove define the several thousands of different alleles found at the classical HLA genes^9^. A complex official nomenclature has been defined for HLA to characterize the extent of its polymorphism. According to this nomenclature, alleles of an HLA gene are defined by the gene name indicating the locus (e.g. HLA-A, -B, -C, -DRB1, -DQB1, -DPB1), followed by a hierarchical numbering system^9^. The 1^st^ field (formerly 2-digit level) defines groups of related alleles. The 2^nd^ field (4-digit) separates alleles within 1^st^ field groups that differ in their protein sequence. Finally, the 3^rd^ and 4^th^ fields define alleles harboring synonymous exonic and non-coding variations, respectively. Additionally, the G-group nomenclature has been introduced in order to merge alleles that have the same nucleotide sequence along the antigen-binding groove and differ in their sequence only outside the groove, thus binding the same repertoire of antigenic peptides.

Past and ongoing pathogen-mediated selection is proposed to be one of the major factors affecting genetic variability at HLA genes^8,10,11^. In addition, HLA genes are associated with various complex genetic disorders in contemporary humans^2^, suggesting a link between historical selection by infectious agents and present prevalence of genetic disorders^12,13^. The recent development of genomic tools for the analysis of ancient DNA (aDNA) provides a unique opportunity to unravel the trajectories of alleles associated with human adaptations to newly introduced or co-evolving pathogens^14,15^. In particular, the investigation of ancient HLA genes in historical populations could shed light on the molecular signatures associated with pathogen-mediated selection and promote the identification of the exact targets of selection. Thus, reliable genotyping of the HLA genes in ancient samples is crucial to answer unresolved questions ranging from human genetics to evolutionary medicine. However, the high density of SNPs^16^ as well as the paralogous organization^17^ of the HLA genes makes their appropriate characterization extremely difficult. Because of the nature of such polymorphisms, SNP-based approaches have very limited applicability, while available NGS-based analysis pipelines rely on deep and homogeneous coverage, as it is readily available from modern DNA samples. DNA molecules extracted from skeletal remains, in contrast, are usually heavily fragmented and degraded through chemical modifications^18^, hence, sufficient genomic coverage of the endogenous DNA can hardly be obtained, adding a further layer of complexity to reliable allele calls within the HLA region. The fragmentation of the aDNA also prevents any of the primer-based amplification approaches (e.g. SSO or SSP) that are commonly used in clinical settings. Thus, to our knowledge, none of the existing HLA genotyping tools have been proved to be suitable for aDNA samples. In this light, the development of an accurate HLA genotyping method applicable to aDNA samples is a prerequisite for studying the evolution of human resistance or susceptibility to pathogens in historical populations.

**Figure 1:**
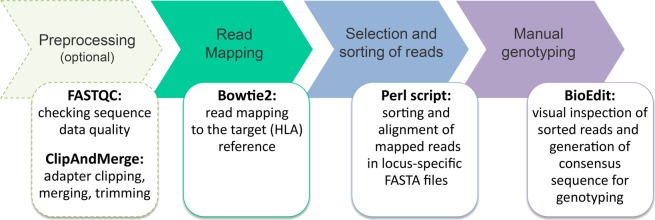
Different steps performed by the TARGT pipeline for HLA genotyping of ancient and modern samples. Preprocessing (optional): after quality control, genomic sequences are pre-processed (adapter clipping, merging and trimming) using ClipAndMerge (version 1.7.3) from the EAGER pipeline^55^. Mapping: performed using Bowtie2^49^ against a comprehensive reference file, containing known 3^rd^ field HLA alleles (following G-group nomenclature). Sorting: mapped reads are grouped by gene specificity and saved into gene-specific FASTA files. HLA genotyping: Sample-specific FASTA files are manually analyzed using BioEdit^50^ to genotype HLA genes in ancient and modern samples.

Here we present a novel aDNA-optimized analysis pipeline for low-coverage and low-quality shotgun sequence data, which we call ‘TARGT’ (Targeted Analysis of sequencing Reads for GenoTyping). The pipeline automatically identifies and sorts target-specific sequence reads from any kind of shotgun short-read sequence data. In principle, it can be used to analyze any targeted region in the genome, but was here applied to the HLA, the most polymorphic genes in the human genome. To identify the specific HLA allele combinations of an individual, the TARGT pipeline combines automated read selection and sorting, with highly repeatable semi-manual filtering and HLA allele identification at up to 3^rd^ field (6-digit) resolution (Fig. 1). After pre-processing and quality control, sequence reads from genomic shotgun sequencing are aligned against a comprehensive reference file containing the exon sequences coding for the peptide-binding groove of all known classical HLA gene variants: class I (HLA-A, HLA-B, HLA-C) and class II loci (HLA-DRB1, HLA-DRB3/4/5, HLA-DQA1, HLA-DQB1, HLA-DPA, HLA-DPB1). The TARGT pipeline is highly versatile; indeed, this step could be adapted and used to target any gene or polymorphic region in the genome by providing a corresponding reference sequence for read selection. Mapped reads are then grouped by gene specificity, and saved into sample- and gene-specific FASTA files. Using a sequence alignment software, the FASTA files can be manually analyzed to genotype individual samples.

While the TARGT pipeline can be applied to any kind of shotgun sequence data, we here present it in combination with a targeted DNA capture approach to analyze HLA polymorphisms in a comprehensive set of historical human samples. Target-enrichment by hybridization, also known as DNA capture, is one of the most widely used approaches for sequencing aDNA, because of its efficiency in increasing the sequence coverage of the endogenous DNA fraction^19–22^. In this work, we used a customized DNA capture approach previously developed for modern DNA and based on sequence information from 8,159 known HLA alleles (available at the IMGT/HLA database^9^)^23^, to enrich a set of DNA libraries from medieval Europeans for the most polymorphic classical HLA class I (HLA-A, -B, -C) and class II (HLA-DRB1, -DQB1, -DPB1) genes. A subset of the obtained HLA class II data has already been analyzed previously in the context of medieval leprosy, yielding a leprosy-associated HLA-DRB1 risk allele^24^. We here assessed the general success of the HLA target-enrichment approach and the overall performance of the TARGT pipeline for both HLA class I and class II genes in the medieval European samples. We then employed a number of different and independent approaches to evaluate the accuracy of the pipeline in producing HLA genotypes. This evaluation includes a comparison with the independent HLA genotyping algorithm OptiType (only available for class I genes, and so far only tested on modern DNA data), with PCR-based results from a specific tag-SNP, and with simulated aDNA sequence data with known HLA alleles. Finally, we explored whether the TARGT pipeline, initially developed for aDNA sequence data, can also be applied successfully to shotgun sequence data from modern populations. For this we applied our approach to a subset of the 1000 Genomes Project samples, for which HLA typing has been performed previously^25^. The TARGT pipeline is freely available for download from SourceForge (https://targt-pipeline.sourceforge.io/).

## Results

### Success of the HLA target-enrichment for historical samples

One of the main purposes of this work was the accurate genotyping of HLA genes in aDNA samples. Thus, a previously described dataset of sixty-eight samples collected from the medieval cemetery of St. Jørgen (Denmark) was used to assess the success of a HLA target-enrichment approach and the subsequent performance of the TARGT pipeline. Owing to different *post mortem* degradation processes over time, DNA molecules retrieved from ancient organisms hold a high level of base pair modifications. Such DNA damages are the source of incorrect incorporation of nucleotides during DNA amplification, and might cause false SNP calling during the final step of sequencing data analysis. The majority of damage-derived miscoding lesions in aDNA sequences are caused by deamination of cytosine into uracil^26–28^. To reduce the rate of ancient DNA errors, treatment with uracil DNA glycosylase and endonuclease VIII (USER mix) is commonly used during library preparation, which generates and cleaves out abasic sites at deaminated cytosines^29^. Having a minimized amount of miscoding lesions, UDG-treated libraries, can lead to higher accuracy of aDNA sequences, and are thus more reliable for downstream population genetic analysis. On the other hand, non-UDG-treated libraries are commonly used to verify ancient DNA authenticity through the investigation of aDNA features like DNA fragmentation and the above described nucleotide misincorporation patterns^27^. We thus performed shotgun sequencing on both UDG-treated and non-UDG-treated libraries for the whole set of sixty-eight individuals. To assess the ancient origin of DNA sequences, the resulting shotgun sequencing data were aligned against the *H. sapiens* reference genome hg38 and postmortem DNA damage signatures evaluated using mapDamage v2.0.6.^30^. The analysis of damage patterns in the final nucleotide of the sequenced fragments revealed misincorporation frequencies of up to 2.6% in UDG-treated and up to 21.9% in non-UDG-treated datasets (Tables S1 and S2). This confirmed that most of the reads mapping to the human reference originate from aDNA fragments. The degree of DNA fragmentation was also explored to further authenticate ancient DNA. The average length of DNA fragments in UDG-treated datasets ranged from 47 to 101 bp (Table S3).

**Figure 2:**
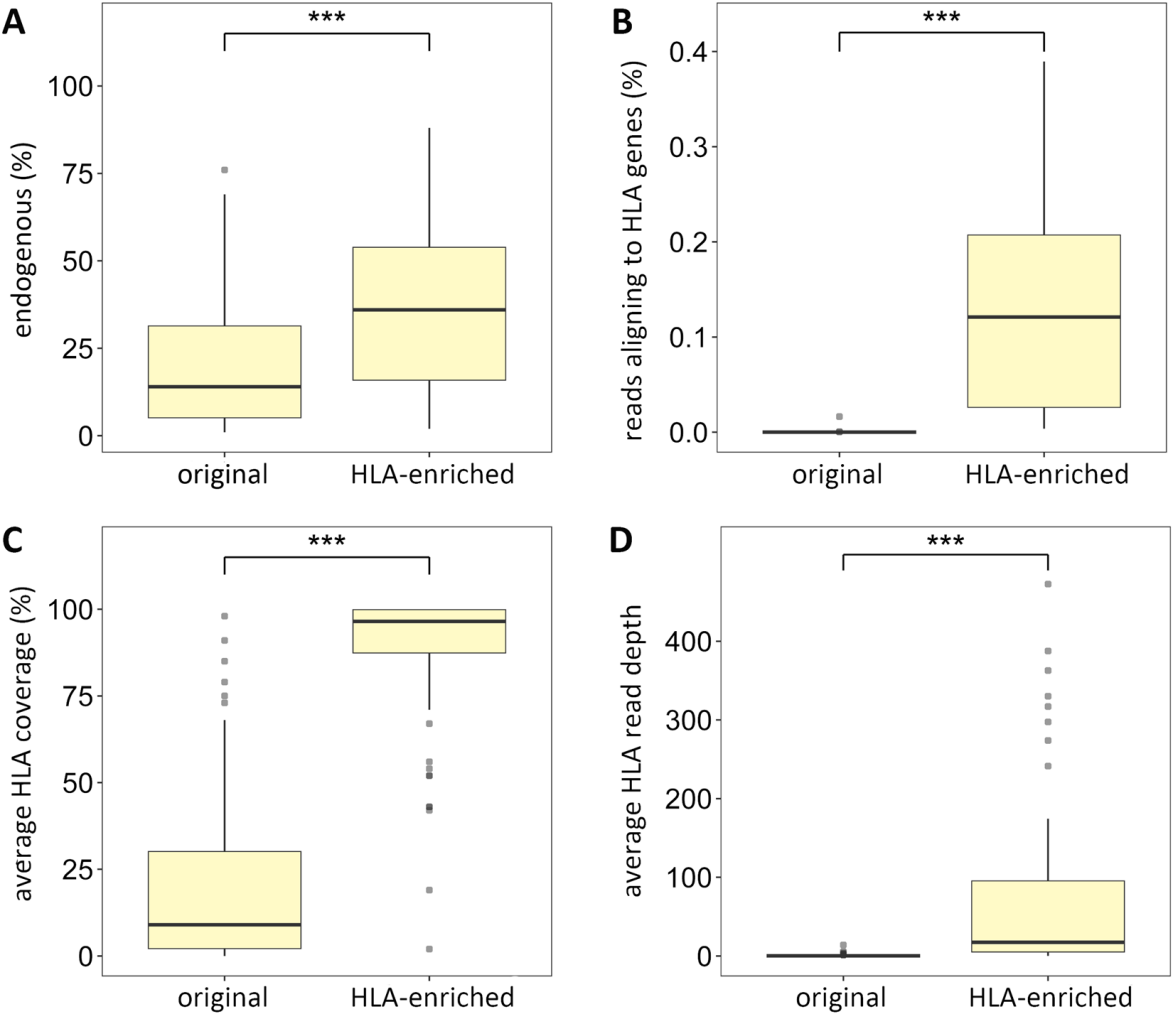
Performance of HLA target-enrichment experiments. Comparison between the median values of (**A**) endogenous DNA content (**B**) percentage of reads aligning to HLA genes (**C**) average HLA coverage and (**D**) average HLA read depth calculated across a subset of 62 aDNA samples before and after performing the HLA enrichment experiments. Significant differences between median values, as derived from Mann-Whitney test, are indicated by horizontal line and asterisks (***p < 0.001). Box plots show median, interquartile range, min-max whiskers and outliers.

In response to the fragmentation and low concentration of endogenous DNA from ancient samples, hybridization capture-based target enrichment can be used to improve the yield of DNA molecules for a specific region of interest. An HLA target-enrichment approach was thus applied to the UDG-treated libraries, from here on defined as HLA-enriched UDG libraries, to investigate the highly polymorphic classical HLA class I (HLA-A, -B, -C) and class II (HLA-DRB1, -DQB1, -DPB1) genes. To evaluate the performance of the capture approach, endogenous DNA content, fold-enrichments as well as average coverage and read depth over the HLA genes were quantified on both original UDG libraries and HLA-enriched UDG libraries for a subset of 62 samples (library comparison for six samples was not possible because of technical problems unrelated to the data quality). The number of reads mapping to the human reference genome ranged from 56,925 to 137,542,840 when considering the original UDG libraries and from 244,945 to 74,490,774 when considering the HLA-enriched UDG libraries (Table S4). The corresponding median endogenous DNA content calculated over the whole set of samples was significantly higher for the HLA-enriched UDG libraries (36%) than for original UDG libraries (14%) (Mann-Whitney, p < 0.001; Fig. 2A and Table 1). The number of reads mapping to the HLA reference ranged from 0 to 8,452 for the original UDG libraries and was higher when considering HLA-enriched UDG libraries, ranging from 359 to 225,013 (Table S4). Consequently, the % of reads aligning to HLA genes calculated over the whole set of samples differed significantly between original (median: 0.0001%) and HLA-enriched UDG libraries (median: 0.1210%) (Mann-Whitney, p < 0.001; Fig. 2B and Table 1). Overall, the HLA enrichment approach performed on the historical samples yielded from 3 to 13,596-fold increases of HLA target sequences compared to the pre-capture condition i.e. the original shotgun sequence data (Table S4). Consistently, the coverage over the HLA genes calculated across the whole set of samples was significantly higher after the enrichment approach (median: 96%) compared to the original shotgun sequence data (median: 9%) (Mann-Whitney, p < 0.001; Fig. 2C and Table 1; for calculation at individual HLA loci see Figure S1 and Tables S5 and S6). Similarly, the read depth quantified for the whole set of samples was significantly lower when considering the UDG shotgun libraries (median: 0.2×) compared to read depth achieved after the HLA target enrichment (median: 17.4×) (Mann-Whitney, p < 0.001; Fig. 2D and Table 1; for calculation at individual HLA loci see Figure S2 and Tables S5 and S6).

**Table 1:** Performance of HLA target-enrichment experiments.

	Sequence data	Min	Max	Median (95% CI)
endogenous [%]	original	1	76	14 (8–25)
	HLA-enriched	2	88	36 (24–42)
reads aligning to HLA genes [%]	original	0.00	0.02	0.00 (0.00–0.00)
	HLA-enriched	0.00	0.39	0.12 (0.05–0.17)
average HLA coverage [%]	original	0	93	7 (5–13)
	HLA-enriched	2	100	97 (94–99)
average HLA read depth (x-fold)	original	0.00	3.98	0.14 (0.11–0.22)
	HLA-enriched	0.12	472.62	18.00 (9.68–68.95)

Endogenous DNA content (%), percentage of reads aligning to HLA genes (%), average HLA coverage (%) and average HLA read depth (x-fold) compared between pre-capture shotgun sequence data (original) and sequence data after HLA enrichment experiments (HLA-enriched) obtained from a subset of 62 historical samples.

### Genotyping of the HLA genes in historical samples

**Figure 3:**
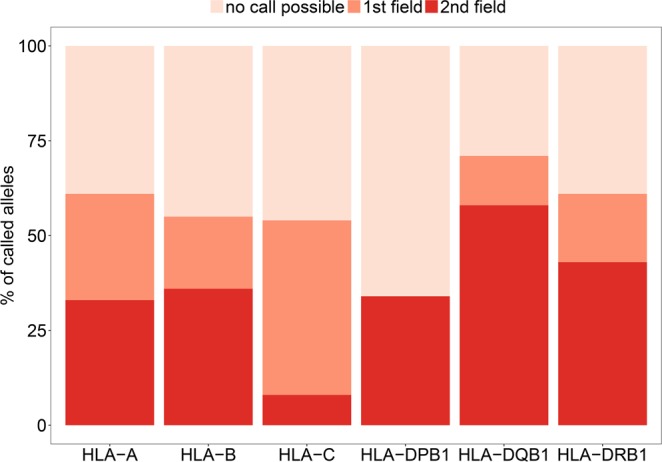
HLA allele call success rate for 68 historical aDNA samples. Percentage of alleles called at each individual HLA locus calculated for the whole set of historical samples (N = 68). Allele calls are reported at two different level of resolution 2^nd^ field (4-digit) and 1^st^ field (2-digit) levels. ‘No call possible’ represents the fraction of cases where the allele call was not possible or allele calls were ambiguous.

To call HLA genotypes from the aDNA samples, all the sequence data generated from UDG-treated libraries were combined for each sample and processed through the TARGT pipeline. The TARGT pipeline allows HLA allele identification at up to 3^rd^ field (6-digit) resolution; however, as most HLA typing tools and HLA genetic studies rely on 2^nd^ field resolution, we are here only reporting results up to this level. Furthermore, when limited read coverage did not allow for 2^nd^ field resolution, usually because several alleles of the same 1^st^ field allele group were equally well supported, the allele call was rounded to that level of resolution (1^st^ field) (Table S7). Of the 136 alleles (2n) investigated at each locus, we were able to call at 1^st^ field level 83 alleles for HLA-A, 75 alleles for HLA-B, 74 alleles for HLA-C, 83 alleles for HLA-DRB1, 96 alleles for HLA-DQB1, and 46 alleles for HLA-DPB1. Of these, the allele call reached the 2^nd^ field resolution for 45 alleles for HLA-A, 49 alleles for HLA-B, 11 alleles for HLA-C, 58 alleles for HLA-DRB1, 79 alleles for HLA-DQB1, and 46 alleles for HLA-DPB1 (Table 2). The success rate calculated across the whole dataset of ancient samples was 56% at the 1^st^ field level and 35% at 2^nd^ field level (for values at each locus see Fig. 3 and Table 3). As expected, a significant positive correlation between coverage and success rate (1^st^ field, Kendall τ = 0.76, p < 0.001; 2^nd^ field τ = 0.76, p < 0.001; Figure S3) as well as between read depth and success rate (1^st^ field, τ = 0.75, p < 0.001; 2^nd^ field τ = 0.75, p < 0.001; Figure S4) was observed, indicating a considerable effect of DNA preservation on allele call success. These associations can explain some cases where poor DNA quality and thus read coverage did not allow for more precise allele calls (Tables 2 and S7). Notably, we found no evidence for more than two alleles at any locus, supporting the notion that the vast majority of DNA fragments in each sample originate from a single individual. These results, together with the examination of misincorporation frequencies and average length of DNA fragments shown above, indicate that the human DNA analysed in each sample is likely to be endogenous.

**Table 2:** Locus-specific allele call success for the 68 historical samples.

	HLA-A	HLA-B	HLA-C	HLA-DRB1	HLA-DQB1	HLA-DPB1
1^st^ field	83	75	74	83	96	46
- of these 2^nd^ field	45	49	11	58	79	46
NA (no call possible)	53	61	62	53	40	90

Number of alleles called at the 1st field level and at the 2nd field level of resolution reported for each locus (2n = 136) for the 68 historical samples.

**Table 3:** Success rate for the 68 historical samples.

	HLA-A	HLA-B	HLA-C	HLA-DRB1	HLA-DQB1	HLA-DPB1	Overall
Success 1^st^ field (%)	61	55	54	61	71	34	56
Success 2^nd^ field (%)	33	36	8	43	58	34	35

Success rate at the 1st field level and at the 2nd field level of resolution, across the 6 investigated genes and overall, for the whole dataset of historical samples.

Because of the high level of linkage disequilibrium (LD) within the HLA region, it has been shown that certain SNPs outside the classical HLA genes are informative about HLA types. Such ‘tag SNPs’ are commonly used to test for association between HLA alleles and disease susceptibility. One example is the T allele at the SNP locus rs3135388, known to be in almost complete LD with the HLA-DRB1*15:01 allele in individuals of European (CEU) ancestry^31^. As reported in the previous study on the sixty-eight medieval samples, this SNP was assayed by PCR and the genotyping results compared to the DRB1*15:01 allele calls obtained with the TARGT pipeline^24^. The study showed perfect correspondence between the tag SNP allele rs3135388-T and the TARGT-based allele calls for the allele DRB1*15:01 where 2^nd^ field allele resolution could be achieved (N = 13), and also with the DRB1*15 call in samples where only the broader 1^st^ field resolution was possible (N = 20, Table S8). That previous study also revealed an interesting observation regarding the specific haplotype structure of the HLA region. Due to the fragmented nature of aDNA, and also due to a lack of intergenic sequence information in the original bait panel of the target capture approach, the haplotype structure of the HLA region cannot be resolved reliably from aDNA. However, the previous study clearly showed a co-occurrence between the allele DRB1*15:01 and the allele DQB1*06:02 (Table S7), suggesting a strong LD between these two loci^24^.

### HLA class I allele call comparison with OptiType

To our knowledge, OptiType^32^ is currently the only HLA genotyping algorithm for which testing of both read depth and read length on prediction accuracy has shown that allele calls appear reliable even for sequence data containing short reads or only a 10x average read depth over the HLA class I loci^32^. These features make OptiType potentially suitable for studying HLA genes from aDNA. However, the algorithm has not been explicitly tested or validated for aDNA and is currently available only for the typing of HLA class I genes. Nevertheless, this tool presently appears to be the only available method to compare with our TARGT pipeline. Thus, to validate our allele calls with an independent approach, a random subset (N = 39) of the historical samples were analyzed using OptiType^32^ v1.3.1, and HLA class I genotype information compared with allele calls from the TARGT pipeline. As OptiType was not designed for low quality data such as usually obtained from aDNA, it has no built-in minimum threshold for data quality, and thus always calls two alleles for a given HLA gene, no matter how spurious the sequence information. For some of the ancient samples, the TARGT approach indicated that low DNA yield and quality, and correspondingly low read coverage, made reliable allele calls impossible at some or all of the investigated HLA genes (Tables S5 and S7). This could be confirmed by visual inspection of the read coverage at the target HLA genes, but OptiType nevertheless produced allele calls also in these cases. However, as it uses the same shotgun sequence data, the reliability of OptiType allele calls are likely to suffer similarly from such data limitations. We therefore excluded those instances from the comparisons, as we were not able to estimate the accuracy of OptiType.

Dividing the number of alleles with identical call in the two approaches by the number of total alleles called, we observed that the two approaches agreed in 93% of the calls. This high agreement rate lends further support to our genotyping approach (Table S9). The number of called alleles that differed between the two approaches (TARGT vs. OptiType) was 12 (7% of all called alleles). One advantage of the TARGT pipeline is that it allows for visual inspection of the supporting sequence reads underlying each allele call. We thus went back to those conflicting allele calls in order to explore the read support for one or the other call. In 5 out of the 12 cases, we found support for our allele call but no support for the call by OptiType. In contrast, in 4 out of the 12 cases, we could not confirm the calls from our approach, but found supporting reads for the allele call by OptiType. In the last three instances, we found that the two different allele calls by the two approaches were both supported and we could not resolve the right allele (Table S9). Note that this evaluation does not include the allele calls by OptiType made with spurious low-quality read data, which are likely to have a significantly higher error rate. Thus, while our approach has a lower success rate in the allele call compared to OptiType, it is likely providing a higher accuracy by avoiding low quality/confidence allele calls. Unfortunately, it is impossible to evaluate this point in more detail as no alternative method is available to obtain the ‘true’ HLA genotypes of ancient samples.

### HLA molecular profile of the historical St. Jørgen samples

As the HLA class II data of these samples have been characterized already in a previous study^24^, we here focus on describing the allele frequency distributions at HLA class I genes (-A, -B and -C; reported in Tables S10 and S11). Twelve distinct allele groups (‘lineages’) at 1^st^ field level of resolution were observed for HLA-A. The A*02 lineage comprises 31% of the total allele pool, with the most common allele A*02:01 seen at a frequency of 0.244. The second most common linage is A*03, with the most common allele A*03:01 also found at a frequency of 0.244. The next two more common linages are A*01 and A*24, for which the most common alleles are A*01:01 (f = 0. 156) and A*24:02 (f = 0.133). The others linages (A*26, A*68, A*11, A*32) are found at frequencies of lower than 10%; while the linages A*29, A*30, A*31 and A*36 as well as the alleles A*02:06, A*31:01, A*32:01 are present as singleton copies. A total of sixteen distinct 1^st^ field level allele lineages were observed at the HLA-B locus, four of which (B*07, B*15, B*44 and B*08) were found at frequencies of greater than 10%. The most common 2^nd^ field HLA-B alleles are B*07:02 (f = 0.204), B*08:01 (f = 0.122), B*40:01 (f = 0.122) and B*44:02 (f = 0.122). The lineage B*42 and the alleles B*27:05, B*35:01, B*35:03, B*45:01, B*55:01 and B*56:01 are found as singleton copies. At the HLA-C locus, a total of eleven distinct lineages at 1^st^ field level of resolution were observed. The three most common lineages (C*07, C*03 and C*04) comprise together over 70% of the total allele pool. The 2^nd^ field level allelic resolution at HLA-C locus was lower as compared with HLA-A and –B loci, nevertheless a total of five distinct 2^nd^ field level alleles were found, with the allele C*07:01 being the most common subtype.

A likelihood ratio test, implemented in Pypop^33^, was used to test the significance of observed linkage disequilibrium (LD) between any two loci (Table S12). The analyses were performed removing individuals with NA at all loci while keeping only allele calls that reached the 2^nd^ field level of resolution. As expect from modern day genetic data, for which high LD has been documented in the HLA region^34–36^, strong linkage signals were also revealed for the historical samples, both within class I and class II as well as between class I and class II loci (Table S12). Locus HLA-A showed significant associations with locus HLA-B (D’ = 0.804; W_n_ = 0.844), HLA-C (D’ = 1; W_n_ = 1), HLA-DRB1 (D’ = 0.703; W_n_ = 0.755) and HLA-DQB1 (D’ = 0.764; W_n_ = 0.709), while no global LD was observed with the HLA-DPB1 locus. Locus HLA-B showed nonrandom associations with all loci: HLA-C (D’ = 0.833; W_n_ = 0.829), HLA-DRB1 (D’ = 0.882; W_n_ = 0.775), HLA-DQB1 (D’ = 0.877; W_n_ = 0.772) and HLA-DPB1 (D’ = 0.829; W_n_ = 0.823). Global LD was revealed also between HLA-C and HLA-DRB1 (D’ = 0.833; W_n_ = 0.882) and between HLA-C and HLA-DQB1 (D’ = 0.875; W_n_ = 0.913). A particularly strong associations was observed between the two adjacent HLA class II loci HLA-DRB1 and HLA-DQB1 (D’ = 0.984; W_n_ = 0.883), while no global LD between the DP locus and the other class II genes were found. Two- and three-locus haplotype frequencies were estimated using the expectation-maximization algorithm. We confirmed the presence of the previously described class II haplotype DRB1*15:01-DQB2*06:02^24^, found at a frequency of 27%. Further common haplotypes were B*07:02-DRB1*15:01 (f = 0.267) and B*07:02-DQB1*06:02 (f = 0.222), suggesting the presence of an extended class I-II haplotype, B*07:02-DRB1*15:01-DQB1*06:02 (f = 0.286). Further extended haplotypes were also observed at appreciable frequencies: A*02:01-DRB1*15:01-DQB1*06:02 (f = 0.125) and B*08:01-DRB1*03:01-DQB1*02:01 (f = 0.143). Several other common two- and three-locus haplotypic associations were observed and are reported in Table S13.

### TARGT pipeline validation on simulated aDNA samples and detection of unknown HLA alleles

**Figure 4:**
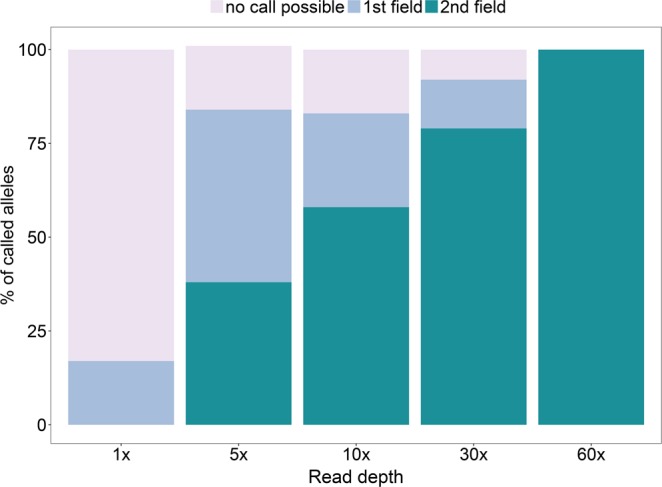
HLA allele call success rate for the simulated ancient samples. Percentage of allele calls calculated across the two investigated loci (HLA-B and HLA-DRB1) and across the set of samples (N = 6) investigated at different read depth (from 1x up to 60×) for a total of 30 simulated ancient samples. Allele calls are reported at two different levels of resolution, 2^nd^ field (4-digit) and 1^st^ field (2-digit). ‘No call possible’ represents the fraction of cases where the allele call was not possible or allele calls were ambiguous.

To assess the efficacy of the TARGT pipeline, we applied it to simulated aDNA sequence data with different read depth levels. Instead of downsampling available human genome data to match the ancient DNA read depth at HLA loci, we took advantage of the gargammel package, a software that can simulate ancient DNA fragments from provided genome sequences^37^. This approach allowed us to consider how both typical aDNA fragmentation and damage pattern can affect reliable allele calls at HLA genes, to verify if and how performance declines with lower read depth, and also to test for the detection of unknown HLA alleles. Seven haplotypes with known but distinct HLA-B and -DRB1 alleles were artificially generated and combined in six different ‘diploid’ combinations (Table S14). Using the program gargammel^37^, typical aDNA fragmentation and damage patterns were introduced in the sequences and the TARGT pipeline tested for increasing read depth from 1x up to 60×, for a total of 30 simulated ancient samples. The HLA allele calls from the TARGT pipeline (here only run on the artificially varied genes HLA-B and HLA-DRB1) were compared to the original known HLA genotypes. If allele calls were not possible, we reported ‘NA’. Allele calls naming several equally well matching 1^st^ field allele groups were considered ambiguous and also reported as ‘NA’. The success rate, defined as the proportion of times an allele call was possible with our approach across the different samples and read depth levels, was 71% at the 1^st^ field level and 57% at the 2^nd^ field level (Table 4). Whenever a call was possible, the allele calls were correct, thus we observed an accuracy rate of 100% for both the 1^st^ field and 2^nd^ field levels (Table 4). However, the 30 ancient simulated samples varied considerably in read depth (from 1× up to 60×), and as expected, the success rate was significantly positively correlated with the read depth. Association between read depth and success rate was observed at both the 1^st^ field and 2^nd^ field levels (Figs. 4 and S5). As the entire set of simulated ancient samples where homogeneous in terms of coverage (i.e. the proportion of covered sites at each locus) we could not test its effect on HLA allele calls in simulated aDNA samples. Intriguingly, we observed that 1^st^ field allele calls were possible already at 1× read depth, while allele calls at 2^nd^ field resolution were obtained starting from a read depth of 5×(Fig. 4). Notably, for some samples the resolution of HLA alleles was not possible even at moderate read depth (30×), underlining that allele call success does not depend exclusively on read depth. Indeed, the specific combination of alleles at each genotype can significantly affect the success in calling the HLA alleles. For instance, highly similar alleles that differ only by a few nucleotides would not be resolved even in samples with high read depth, if those few nucleotide positions happen to not be covered by any read. On the other hand, if a particular region that differentiates the two alleles is covered by only a few reads, an accurate allele call might be possible even if the rest of the gene is not covered at all. We further tested if the TARGT pipeline can detect unknown alleles, such as HLA alleles that were present in human history but no longer exist in modern populations, or extremely rare alleles that are not represented in the HLA reference database. To do so, we introduced three point mutations in two out of the seven artificially generated haplotypes. All of them were well detected starting from a read depth of 5× (Table S14). These results confirm that the mapping of shotgun sequence reads to a reference file containing known HLA alleles, an inherent component of the TARGT pipeline, does not prevent the detection of novel alleles.

**Table 4:** Success rate and accuracy rate for simulated ancient DNA samples.

	HLA-B	HLA-DRB1	Overall
Success 1^st^ field (%)	62	80	71
Success 2^nd^ field (%)	50	64	57
Accuracy 1^st^ field (%)	100	100	100
Accuracy 2^nd^ field (%)	100	100	100

Success and accuracy rate of HLA allele calls, at the 1st field level and at the 2nd field level of resolution, across the 2 investigated genes and overall, for the simulated ancient samples.

### Pipeline validation on 1000 Genomes Project samples

The TARGT pipeline was initially developed with the aim to define HLA alleles from aDNA sequence data. However, since shotgun low-coverage resequencing of modern population samples is becoming more and more common, our pipeline might also be useful in that context. In order to test whether the TARGT pipeline can also be successfully applied to shotgun sequence data from modern populations, we genotyped the classical HLA genes in a diverse subset of individuals from the 1000 Genomes Project, for which HLA genotype information has previously been published (N = 31; Tables S15–S17). The majority of the allele calls obtained through the TARGT pipeline were consistent with the ones obtained through independent PCR-based genotyping in Gourraud *et al*.^25^, as evidenced by the high agreement rate of 96% (Tables S16–S18). The total number of called alleles that differed between the two approaches was five (4%). After careful inspection of the available read data, we found supporting evidence for the allele call by Gourraud *et al*. in three out of the five cases. In contrast, in two of the five cases, the data did not support the call by Gourraud *et al*. but instead clearly confirmed our allele calls, showing that the TARGT pipeline can rectify erroneous calls from PCR-based HLA genotyping. The success rate for allele calling obtained with the TARGT pipeline was 100% at the 1^st^ field level and 90% at the 2^nd^ field level. Indeed, the majority of the called alleles could be defined at the 3^rd^ field level, using the G-group nomenclature (Table S18). When looking at individual loci, a higher success rate was achieved for allele calls at the HLA-DRB1 locus compared to HLA-B (Table 5). The accuracy rate of the called alleles was 99% at 1^st^ field (2-digit) resolution, and 97% at 2^nd^ field (4-digit) resolution. Also in this case, higher accuracy was obtained for the allele calls at the HLA-DRB1 locus in comparison to the HLA-B, at both 1^st^ field and 2^nd^ field resolution (Table 5).

**Table 5:** Success rate and accuracy rate for the 1000 Genomes Project samples.

	HLA-B	HLA-DRB1	Overall
Success 1^st^ field (%)	100	100	100
Success 2^nd^ field (%)	84	95	90
Accuracy 1^st^ field (%)	95	100	99
Accuracy 2^nd^ field (%)	95	100	97

Success and accuracy rate of HLA allele calls, at the 1^st^ field level and at the 2^nd^ field level of resolution, across the 2 investigated genes and overall, for a diverse subset (N = 31) of the 1000 Genomes Project samples.

## Discussion

The investigation of HLA genes in ancient and modern human populations is of great interest to answer unsolved questions in the fields of biomedicine and evolutionary biology. As independent targeted HLA genotyping is very costly, the possibility to obtain HLA genotypes from the low-coverage shotgun sequence data that is now routinely generated in population genomic studies would be highly advantageous. We therefore present here the novel TARGT pipeline for HLA genotyping from low-quality shotgun data, and evaluate its accuracy for ancient and modern DNA samples. The pipeline can be applied directly to shotgun sequence data or can be combined with a target enrichment approach.

Widely used in the aDNA field, the target-enrichment approach can effectively recover endogenous DNA fractions of interest, targeting SNPs^38,39^, whole chromosomes^21,40^, mitochondrial^19,41,42^ or nuclear genomes^20,22,43,44^.

We here applied a customized DNA capture approach^23^ to enrich a defined set of sixty-eight aDNA libraries for the classical HLA class I (HLA-A, -B, -C) and class II (HLA-DRB1, -DQB1, -DPB1) genes. Endogenous DNA content, percentage of reads aligning to HLA genes as well as coverage and read depth over the HLA genes were compared between shotgun libraries and HLA-enriched libraries (Fig. 2 and Table 1). The comparison between pre-capture shotgun sequence and sequence data obtained after HLA target enrichment yielded from 3-fold to 13,596-fold increases of HLA target sequences and clearly showed the efficiency of the HLA enrichment approach in increasing the number of reads mapping to the HLA genes of interest. These results highlight the advantage of the HLA target enrichment for studying HLA polymorphisms in ancient human populations.

We then applied the TARGT pipeline (Fig. 1) to the aDNA sequence data and evaluated the resulting HLA allele calls. The success rate was variable across the different loci with the highest success rate obtained at the HLA-DQB1 locus, followed by HLA-A, HLA-DRB1, HLA-B and HLA-C; while the HLA-DPB1 locus showed the lowest success rate (Fig. 3 and Table 3). As the achieved average coverage was comparable across the different loci (Figure S1 and Table S6), the differential success rate is unlikely to be due to an unbalanced bait design. The general differences in both the allelic diversity as well as in the extent of sequence divergence between alleles at the different HLA loci, together with the uneven distribution of nucleotide diversity along the investigated exons, are more plausible explanations for the observed variable resolution across loci. Furthermore, DNA preservation of individual samples can affect allele call success as shown by the strong association between success rate at each sample and both read depth and coverage. Thus, the observed success rate calculated for the historical dataset (56% at 1^st^ field resolution; 35% at 2^nd^ field) cannot be generalized for other ancient samples, as it will likely vary depending on the spatial and temporal scales investigated as well as on the degradation of the underlying DNA.

The allele calls obtained with the TARGT pipeline were evaluated using an independent approach, the software OptiType^32^, which is currently available only for the typing of HLA class I genes. The comparison revealed a high level of agreement between the two approaches (93%) for the HLA genotyping of class I alleles. Moreover, the presence of the most frequent variant at one of the class II genes, the allele DRB1*15:01, was supported by the independent detection of a specific tag-SNP in a previous study^24^. Both comparisons provided further evidence for the accuracy of our allele calls, supporting the validity of the TARGT pipeline.

Our pipeline was also evaluated on simulated aDNA sequence data with distinct but known HLA-B and -DRB1 alleles. Here we observed a success rate of 71% at the 1^st^ field level and 57% at 2^nd^ field level of resolution, both with an accuracy of 100% (Table 4). We tested the pipeline for increasing read depth (from 1× up to 60×) and observed also in this case a strong association with success rate. However, we noticed that in some cases alleles could be called at very low read depth: some 1^st^ field alleles could be called already at 1x depth, while calls at 2^nd^ field resolution were obtained starting from 5× read depth (Fig. 4). In contrast, in a few instances, the resolution of HLA alleles was not possible even at moderate read depth (30×), suggesting that read depth is not the only factor influencing the allele call success. Whilst additional sequence reads in terms of read depth allow the identification of sequencing errors and can provide support for specific allele calls, informative overlap among reads to build a consensus sequence of sufficient length is also essential to obtain reliable HLA genotypes. Indeed, the proportion of covered sites at each locus together with the specific combination of alleles can significantly affect the success in calling HLA alleles. For instance, if the alleles of a given genotype differ only in positions that are not covered by any reads, a full allelic resolution will be impossible, even if the rest of the sites are covered at high depth. With the allele call success depending on various properties of a given DNA sample, it would be inappropriate to define a default threshold of read depth or coverage for applicability of the TARGT pipeline, especially for ancient samples. However, the advantage of our semi-manual approach is that the experimenter receives direct visual feedback about the quality and quantity of the sequence data and can make an informed decision about the reliability of any allele call. The possibility of visual inspection of the raw data is particularly crucial in case of aDNA samples; this feature sets our pipeline apart from other more automated approaches (presently only available for modern DNA), some of which will always provide an allele call, no matter how spurious and ambiguous the underlying sequence data. During the evaluation process on simulated aDNA sequences we also observed that the HLA allele calls obtained with the TARGT pipeline achieved high accuracy even in samples whose HLA alleles are not included in the original HLA reference file. These results show that our new approach has the potential to detect unknown alleles, such as extremely rare alleles that are currently absent in the HLA reference database or alleles that were present in the past but no longer exist in modern populations.

We then also applied our pipeline to a subset of the 1000 Genomes Project samples in order to test its applicability for shotgun sequence data of modern DNA from population genomic projects. Modern DNA usually exhibits no degradation or extensive fragmentation, thus yielding much longer sequence reads and more even coverage, making allele calling much easier. Consequently, we observed a much higher success rate (100% at the 1^st^ field; 90% at the 2^nd^ field level of resolution) compared with both the empirical and simulated ancient datasets (Table 5). These results, together with the high accuracy rate observed (99% at the 1^st^ field level; 97% at 2^nd^ field level), suggest that the TARGT pipeline, originally developed to define HLA alleles in aDNA samples, can also be successfully applied to shotgun sequence data from modern DNA.

The pipeline includes a pre-processing part with several automated steps essential for the analysis of any next-generation sequencing data: quality control, adapter clipping, and merging of paired reads (Fig. 1). This part is optional and can be applied if raw sequence data is to be used for HLA analysis. However, if WGS/WES shotgun data has already been quality-checked and trimmed for other purposes, this can also be used directly as input data for the TARGT pipeline, which would then start with the mapping and sorting steps (Fig. 1). Important in the latter case is the awareness that duplicate reads will likely have been removed during quality filtering. For modern sequence data with decent coverage, this might not be a problem, but for data from aDNA, information about read abundance can be an important parameter during allele calling. In this case, it might be advisable to start the pipeline on the raw data instead, and redo the quality filtering specifically with the TARGT pipeline, not discarding duplicate reads. For the mapping step, it is also recommended to carefully consider the optimal mismatch threshold for successful mapping of reads to the HLA reference. A very stringent threshold (e.g. 0% mismatch allowed) will lead to individual reads mapping to fewer HLA loci/alleles in the reference and thus provide less ambiguous read data for allele calling. However, such a stringent threshold might also lead to missing of novel/unknown alleles in a sample, as their specific reads might not map well enough to any known allele and thus would be thrown out. This trade-off should be considered for each given dataset/project, and it might be advisable to run the pipeline multiple times with different thresholds in order to detect the presence of unknown alleles. The default threshold of 1% mismatch balances this trade-off and appears to be a good starting point for most datasets in our experience. The subsequent visual inspection of HLA sequence reads is a critical step for the correct calling of HLA alleles, especially with aDNA samples where miscoding lesions and fragmentation, in combination with the high density of SNPs and paralogous sequences naturally present in the HLA region, can easily lead to incorrect allele calls. The manual identification of HLA alleles allows detection of small differences between alleles and was successful in detecting novel HLA variants in our evaluation. Furthermore, discrepancies between different PCR-based methods routinely used for HLA typing have been observed^45^, highlighting that inaccurate HLA allele calls could be a problem also in case of modern samples. In this context, we have shown that our approach successfully identifies incorrect genotypes and thus allows validation of HLA allele calls from other, even more established, methods.

Despite the advantage of reaching high accuracy by preventing low quality/confidence allele calls, we recognize that our approach can be time-consuming and that the visual inspection of the sorted reads might depend on the experience of individual researchers. However, in a previous study on medieval leprosy victims, it was shown that the results of the TARGT pipeline, including the manual allele call by different researchers, were highly reproducible, reaching >99% reproducibility at the nucleotide level^24^. Eventually, every genotyping approach has its advantages and disadvantages, and it has proven difficult to establish a single best-performing method among the growing number of available computational tools for HLA typing^45^. In such a situation, the most appropriate approach would be to use consensus information that integrates results from different complementary methods. In this context, the TARGT pipeline has a true advantage by providing an independent, non-automated allele call that includes visual inspection of the underlying sequence data and intuitive feedback about the reliability of the call (also with regard to calls from other methods that use the same sequence data).

Our results show that the TARGT pipeline is an accurate method for HLA genotyping in case of low-coverage shotgun sequence data. The pipeline also allows for the detection of unknown alleles that are not included in the original HLA reference database. The observed solid performance demonstrates that TARGT is a reliable approach to accurately genotype HLA genes in ancient and modern DNA samples. The pipeline has already been applied successfully to a dataset of medieval European samples, associating HLA variability with susceptibility to leprosy^24^, and indicating its applicability to study the evolution of human resistance or susceptibility to pathogens in historical populations. In addition, the pipeline could be employed to explore HLA allele frequency changes through time, when temporal sample series are available^46^, thus providing a deeper understanding of HLA genetic variation through human history.

## Methods

### HLA typing from shotgun sequence data (TARGT pipeline)

#### HLA reference file for read mapping

A key component of the TARGT pipeline is a comprehensive HLA reference file containing all known nucleotide sequence variants of the exons coding for the peptide-binding groove of the classical class I (exons 2 and 3; HLA-A, HLA-B, HLA-C) and class II HLA genes (exon 2; HLA-DRB1, HLA-DQA1, HLA-DQB1, HLA-DPA, HLA-DPB1). This reference allows differentiation of HLA alleles at up to 3^rd^ field (6-digit) resolution using the G-group nomenclature. The G-group nomenclature groups together HLA alleles whose peptide-binding domains are identical at the nucleotide level (and thus also at the protein level^47^). The reference also contains corresponding sequence variants for non-classical HLA genes, to avoid mis-mapping and misidentification of reads, due to paralogous sequence similarity. To build this reference, nucleotide coding sequences were downloaded from the IMGT/HLA database^9^ (accessed 28 July 2015) for the following loci: HLA-A, -B,-C, -E, -F, -G, -H, -J, -K, -L, -U, -V, -DQA1, -DQB1, -DRA, -DRB1, -DRB3, -DRB4, -DRB5, -DRB6, -DRB7, -DRB9, -DPA1, -DPB1. Exon sequences of HLA-DQB2 from the human reference genome (not represented in the IMGT/HLA fasta files), was also included, again to preclude misidentification of its reads as HLA-DQB1 variants. Alignment of selected nucleotide sequences was then performed individually for each locus using the program muscle^48^ v3.8. Gaps caused by rare non-functional alleles were removed as well as overhangs upstream and downstream of the exons of interest. Redundant sequence variants that are identical within the exons of interest were also removed (following the G-group nomenclature). One hundred nucleotides (Ns) were introduced upstream and downstream of all sequences, while 20 Ns were introduced between exon 2 and 3 for class I loci in order to allow for mapping of reads crossing the exon-intron border, as the intron sequences for most alleles are not available from the IMGT/HLA database. All aligned sequences were combined into one FASTA file, which was finally indexed using Bowtie2 to produce the final HLA reference file.

#### Read mapping

The standard input for the TARGT pipeline is qc-filtered and adapter-trimmed short-read sequence data in fastq format. The first step maps the sequence reads of the sample to the HLA reference file using Bowtie2^49^ v2.2.7 in local alignment mode. Bowtie2 allows mapping of both merged reads and separate paired-end reads. By using the ‘-a reporting mode’, we allow each read to map against multiple alleles in the reference, which is crucial because of the expected ambiguous mapping of most reads. To achieve a maximum mismatch threshold of 1%, the minimal alignment score was set to –score-min L,0,0.99, while keeping the local alignment matching bonus setting of –ma 1, and the maximum (MX) and minimum (MN) mismatch penalties equal to –mp 0,0. Allowing for 1% mismatch represents a balanced trade-off between mapping sensitivity and specificity, while at the same time enabling the identification of unknown alleles (not present in the reference file). However, this mismatch threshold can be varied, and should be carefully chosen with regard to the specific study question (see discussion).

#### Automated read sorting

Output from mapping with Bowtie2 contains reads that aligned best exactly one time to the HLA reference file as well as ambiguous reads i.e. reads that map equally well to multiple alleles in the reference. Such ambiguous reads can map to multiple alleles of the same locus and to alleles from different loci. In the latter case, multiple instances of the same read sequence, one for each distinct mapping locus, are stored in the resulting alignment. The mapping information from the Bowtie2 output is processed with a Perl script (included in the pipeline scripts on Sourceforge). During this processing procedure, identical reads, representing PCR duplicates of the same DNA fragment, are collapsed and their absolute frequency is noted. For each read, the number of duplicates as well as the mapped locus name(s) and number of alleles per locus are stored in the read’s fasta tag. Read sequences are then grouped by locus and saved into a FASTA file in a specific format: sequences are ordered, in ascending order, according to the number of genes they map to and then sorted by the starting position within the corresponding locus so that they follow the sequence orientation along the locus of interest (Figure S6). The thus generated locus-specific FASTA files can then be inspected for manual allele calling using a sequence alignment editor.

#### Manual read sorting and allele calling

For the manual read analysis and allele calling, we use the freely available and versatile sequence alignment editor BioEdit^50^ v7.2.565, which permits visual inspection and manipulation of sequence reads. However, in principle, any sequence alignment editor can be used for this purpose as long as it facilitates the steps described here. The locus-specific FASTA files generated by the above Perl script were opened in BioEdit and consensus sequences of the allele combinations present in each sample were generated. First, by visually inspecting sequence identity among reads, true SNPs (identifying the true alleles) were distinguished from PCR/sequencing errors. Sequence reads representing the possible alleles were then identified and sorted into blocks of reads belonging to the same allele (Figure S6). Here, reads were prioritized that map uniquely to the locus of interest, and/or that were represented by multiple exact PCR duplicates (less likely to represent PCR/sequencing errors). Overlapping reads that shared the same combination of variations were collapsed into a consensus sequence. To identify matching alleles, consensus sequences were compared to a reference alignment of all known 4-digit alleles of the corresponding HLA locus. Such comparison was performed first focusing on an established set of ‘common and well-documented’ HLA alleles^51^. If the consensus sequences perfectly matched one or more alleles from that set, we did not look for additional matches in the rarer alleles; otherwise, the full set of all known alleles of that locus was screened for best-matching sequences. The full nucleotide sequence of the identified allele was finally compared to the read alignment to confirm that the allele call was indeed supported by all high confidence reads. In case of several equally well matching alleles belonging to the same two-digit allele group (e.g. because of incomplete coverage), only the 1^st^ field (two-digit) allele name was reported.

### Sequence data from historical samples

#### Historical samples

The human skeletal remains whose DNA was analyzed in this study were obtained from the medieval cemetery of St. Jørgen/Denmark. Sixty-eight individuals were considered for this study, ranging in age from 1270 to 1536 AD, with most of the individuals falling between 1270 and 1400 AD^24^. Sample processing, DNA extraction and DNA library preparation have previously been described in Krause-Kyora *et al*.^24^. DNA extractions and pre-PCR steps were performed in clean room facilities dedicated to aDNA research, following the guidelines on contamination control in aDNA studies^52–54^. For each sample, two different double-stranded DNA sequencing libraries (UDG-treated and non-UDG-treated) were prepared. Both UDG-treated and non-UDG-treated libraries underwent paired-end shotgun sequencing carried out on the Illumina HiSeq 2500 (2 × 125 bp) and HiSeq 4000 (2 × 75 bp) platform at the Institute of Clinical Molecular Biology, Kiel University, using the HiSeq v4 chemistry and the manufacturer’s protocol for multiplex sequencing.

#### HLA target-enrichment for historical samples

UDG-treated libraries were enriched for DNA from the classical class I (HLA-A, HLA-B, HLA-C) and class II HLA genes (HLA-DRB1, HLA-DQA1, HLA-DQB1, HLA-DPA, HLA-DPB1), using a custom bait library designed by Wittig *et al*.^23^. The HLA capture probes have been originally created considering the full list of available cDNA and gDNA sequences from the IMGT/HLA reference database^9^ (i.e. 8,159 alleles), which resulted in a total of 16,351 distinct RNA baits, covering a cumulative target genomic sequence of 215.5 kb^23^. The in-solution targeted capture has been performed using the SureSelectXT Target Enrichment System (Illumina) for the Illumina paired-end multiplexed sequencing library (version B4, August 2015). For each capture reaction, up to four UDG-treated libraries have been pooled. The hybridization reaction required 800 ng of library DNA per pool in a volume of 3.4 µL. As the UDG-treated libraries were already indexed during library preparation, the 12 cycles of post-capture PCR was performed using 1 µL of each IS5 and IS6 primers (100 µM). According to the protocol, the resulting amplified captured libraries were purified using the AMPure XP beads, while quality assessment was performed on the Agilent 2100 Bioanalyzer with the High Sensitivity DNA Assay. Finally, sequencing was done on the Illumina HiSeq 4000 (2 × 75 cycles) platform at the Institute of Clinical Molecular Biology, Kiel University, using the HiSeq v4 chemistry and the manufacturer’s protocol for multiplex sequencing.

#### Data preprocessing for historical samples

HTS data sets generated for the sixty-eight individuals from St. Jørgen were pre-processed (adapter clipping, merging, trimming) using ClipAndMerge (version 1.7.3) from the EAGER pipeline^55^. During the adapter clipping step, adapters were excluded when present in the sequence, while reads with fewer than 25 nucleotides after adapter clipping or containing only adapters sequences were removed. In the merging step, all remaining paired reads were merged with a minimum overlap of 10 nucleotides and at most 5% mismatches in the overlap region. In the final quality trimming phase, all nucleotides with Phred scores smaller than 20 were trimmed from the 3’ end of each read, while sequences shorter than 25 nucleotides after quality trimming were removed. In order to evaluate postmortem DNA damage signatures, using mapDamage v2.0.6^30^, shotgun sequencing data from both UDG-treated and non-UDG-treated libraries were aligned against the *H. sapiens* reference genome hg38 (GRCh38) using Bowtie2^49^ v2.2.7, in a semi-global alignment mode and with default parameters, as described in Krause-Kyora *et al*.^24^. Read duplicates were not removed during the pre-processing and quality filtering steps, as read redundancy information is used during manual HLA allele call for identifying sequencing artifacts. Endogenous DNA content, percentage of reads aligning to HLA genes as well as coverage and read depth over the HLA genes were quantified on both the original UDG shotgun libraries and the HLA-enriched UDG shotgun libraries. Endogenous percentage was measured by the proportion of reads mapping to the human reference genome over the total amount of reads. Percentage of reads aligning to HLA genes was calculated as the proportion of reads mapping to the HLA reference over the total amount of reads. Fold-enrichment was calculated by dividing the number of on-target reads, i.e. reads mapping to the HLA reference, from HLA-enriched libraries by the number of on-target reads from pre-capture shotgun libraries; when the denominator was 0 the number of on-target HLA reads from enriched libraries has been assigned. Coverage was calculated as the proportion of covered sites at each locus. Because of the extensive number of reads mapping to multiple HLA loci, read depth (i.e. number of times a base at a given locus is sequenced) has been calculated weighing the read length for the number of loci that each read would map to. Also, to exclude reads containing PCR/technical duplicates, we considered each read with the same starting and ending position only once. Average HLA coverage and average HLA read depth are the mean of coverage and read depth calculated across the 6 investigated class I (HLA-A, -B, -C) and class II (HLA-DRB1, -DQB1, -DPB1) genes.

#### Statistical analysis for historical samples

Allele frequencies in historical samples for class I genes (HLA-A, -B and -C) at the 1^st^ field level and 2^nd^ field level of resolution were obtained by direct counting. Pairwise estimates of nonrandom associations between each pair of HLA loci, i.e. linkage disequilibrium (LD), as well as frequent haplotypes in high LD were determined using PyPop^33^ with defaults settings. PyPop is a software pipeline, originally developed for the analysis of highly polymorphic human leukocyte antigen (HLA) data, and thus useful to perform genetic statistics from multilocus genotype. Overall LD between pairwise HLA genes was defined through two measures. The first one is the normalized Hedrick’s D’ statistic (D’) which weights the LD contribution of specific allele pairs by the product of the allele frequencies at each locus^56^. The second one is the Cramer’s V statistic (W_n_) which defines the normalization between zero and one of the chi-square statistic for deviations between observed and expected haplotype frequencies^57^. The normalized LD values (D’ and W_n_) range between 0 and 1. The permutation distribution of the likelihood-ratio test has been used to test the significance of the overall LD between pairwise HLA genes^58^. Two- and three-locus haplotype frequencies were estimated from the ancient genotypic data, using the iterative expectation-maximization (EM) algorithm^59,60^. The analyses were done removing individuals with NA at all loci while keeping only allele calls that reached the 2^nd^ field level of resolution.

#### Allele call comparison to OptiType pipeline

To compare the allele call results obtained with the TARGT pipeline with an independent method, sequence data of the historical samples were also analyzed using OptiType^32^ v1.3.1. The OptiType pipeline in its present form allows only analysis of HLA class I loci. Results of the genome-wide alignment against the human reference were used as input and FASTQ files were generated from aligned BAM files using samtools. OptiType was then applied in DNA mode with default settings.

### Sequence data from simulated aDNA samples

To validate our HLA genotyping pipeline, we generated simulated aDNA data from genomes with known HLA variants. For this, we first created seven unique MHC haplotypes containing known HLA-B and -DRB1 alleles. The nucleotide sequence of the classical MHC region (chr6: 29,640,000–33,120,000)^61^ was downloaded from the UCSC Genome Browser, using the human reference genome GRCh38. The exons forming the variable region in the peptide binding groove (i.e. exon 2 and 3 for the HLA-B locus and exon 2 for the HLA-DRB1 locus) of known alleles were first aligned and then manually edited using BioEdit^50^; thus creating haplotypes with different alleles from the reference genome. Additionally, ‘de-novo mutations’ were introduced in two out of seven haplotypes: in the first haplotype, a point mutation was introduced at each locus (HLA-B and -DRB1); while in the second haplotype a point mutation was introduced only at the HLA-DRB1 locus. The 7 unique HLA haplotypes were then combined in six different diploid combinations of heterozygous genotypes (Table S14). Typical bias observed in aDNA samples (fragmentation and damage patterns) were then introduced using the program gargammel^37^. For each genotype, we created five aDNA paired-end read datasets with increasing read depth (1×, 5×, 10×, 30×, 60×), for a total of 30 simulated aDNA samples. In simulating DNA fragmentation, fragment size was calculated considering the average fragment length observed in the set of medieval European samples tested in this study. In the same way, deamination patterns and base content profile were also obtained from one of the investigated ancient samples (G507). One of the advantages of the capture approach is that it can drastically reduce the extensive microbial contamination often present in ancient samples; we thus did not introduce microbial contamination while simulating the ancient HLA regions. Ideally, the assessment of the ancient origin of DNA sequences can be evaluated, and ancient samples suspected to contain human contamination should be excluded from the analysis. Because of that, human contamination was also not introduced in our simulated samples. To avoid any observer bias in the allele call of simulated aDNA samples, the HLA genotyping was performed by two independent researchers that were not aware of the specific alleles introduced in the simulated samples.

### Modern sequence data from 1000 Genomes Project

In order to assess the applicability of our pipeline for shotgun sequence data from modern samples, we used whole-exome shotgun sequence data from the 1000 Genomes Project^62^. Paired-end sequencing datasets from 31 individuals of diverse ancestry (8 Africans, 8 East Asians, 7 Americans, and 9 Europeans) were downloaded from the 1000 Genomes Project database (phase 3) (Table S15). Only samples with available SBT-based HLA genotype information published in Gourraud *et al*.^25^ were included (Tables S16 and S17).

### Evaluation of HLA allele calling pipeline

To assess the reliability of the TARGT pipeline, three different measures were defined. The success rate quantifies the proportion of cases where an allele call was possible, in both the empirical datasets (historical samples and 1000 Genomes samples) as well as in the simulated aDNA samples. It was defined as the ratio between the number of called alleles provided by our approach and the total number of the alleles assayed (two per locus and sample). The success rate reported here can be considered conservative since ambiguous results (i.e. allele call with several equally well matching alleles belonging to different two-digit allele groups) were reported as NA (allele call ‘not available’). The measure of agreement was used to compare the allele calls from two independent methods. It was calculated as the proportion of identical alleles typed using the two approaches over the total number of the alleles called, thus excluding alleles for which allele call was not possible in one or both approaches. The accuracy rate was used to assess the confidence of HLA genotypes provided with our approach, when HLA alleles were known a priori, as in the case of the simulated ancient samples, or when HLA alleles have been previously typed with different approaches in the case of 1000 Genomes samples. It was calculated as the proportion of correctly called alleles over the sum of correctly and incorrectly called alleles; also in this case non-possible allele calls were excluded.

## Supplementary information

Supplementary information.

## Data Availability

Pipeline scripts together with instructions and ancillary files are freely available online (https://targt-pipeline.sourceforge.io/). Sequence data were obtained from Krause-Kyora *et al*.^24^ and are accessible in the European Nucleotide Archive under accession no. ERP021830 (https://www.ebi.ac.uk/ena/data/view/PRJEB19769).
