# Supplementary material for: Targeted analysis of polymorphic loci from low-coverage shotgun sequence data allows accurate genotyping of HLA genes in historical human populations
Source: Sci Rep. 2020 Apr 30;10:7339. doi: 10.1038/s41598-020-64312-w (PMC7193575; doi:10.1038/s41598-020-64312-w)
Supplement: Supplementary file 1 — Supplementary information. [file 41598_2020_64312_MOESM1_ESM.pdf]

Supplementary information for

Targeted analysis of polymorphic loci from low-coverage shotgun sequence data allows accurate genotyping of HLA genes in historical human populations

Federica Pierini[1,†], Marcel Nutsua[2], Lisa Böhme[2], Onur Özer[1], Joanna Bonczarowska[2], Julian Susat[2], Andre Franke[2], Almut Nebel[2], Ben Krause-Kyora[2] and Tobias L. Lenz[1,*]

* Corresponding author: Tobias L. Lenz, lenz@post.harvard.edu

Supplementary Data include:

Figures S1-S6

Tables S1-S18

Figure S1. Median of coverage compared between pre-capture shotgun sequence data (original) and sequence data after HLA enrichment experiments (HLA-enriched) across a subset of 62 historical samples and reported for the individual HLA genes. Significant differences between median values, as derived from Mann-Whitney test, are indicated by horizontal line and asterisks (***p < 0.001).

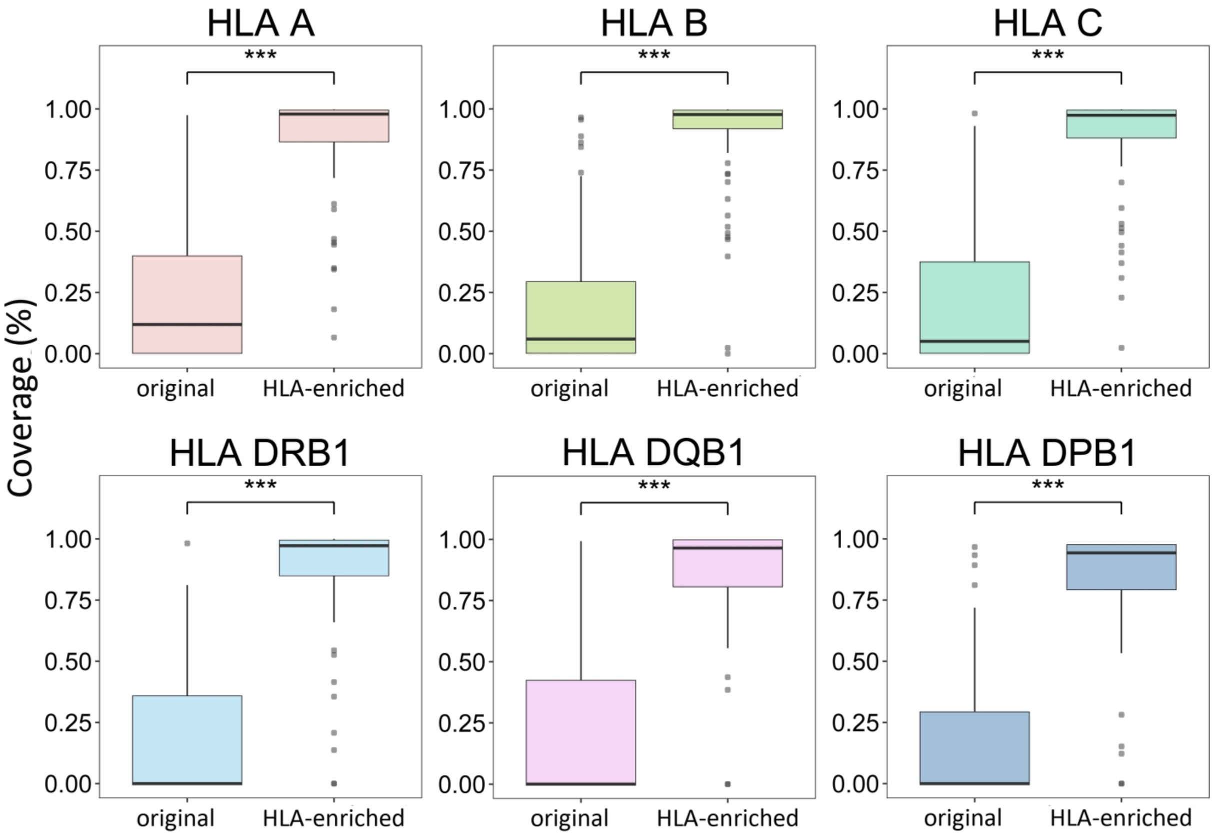

Figure S2. Median of read depth compared between pre-capture shotgun sequence data (original) and sequence data after HLA enrichment experiments (HLA-enriched) across a subset of 62 historical samples and reported for the individual HLA genes. Significant differences between median values, as derived from Mann-Whitney test, are indicated by horizontal line and asterisks (***p < 0.001).

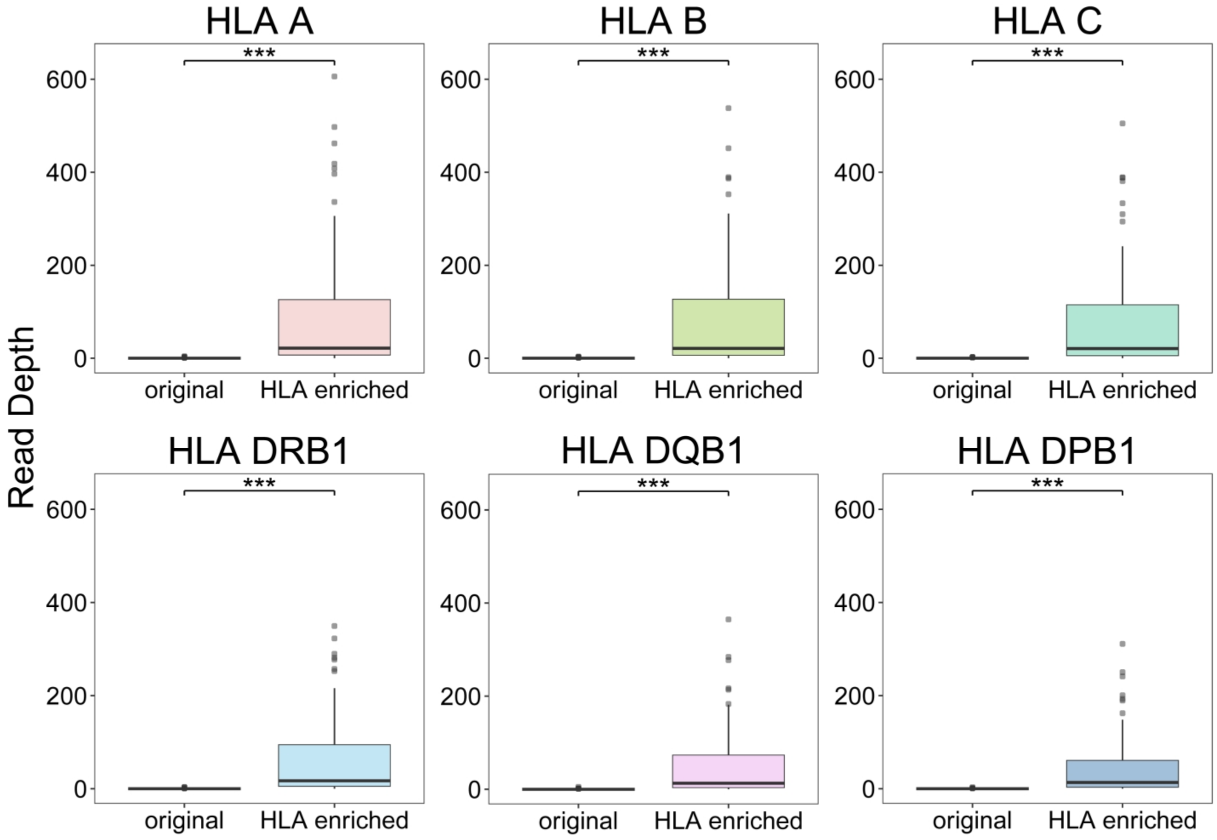

Figure S3. Correlation between average coverage over the HLA genes and success rate at two different levels of resolutions (A: 1st field; B: 2nd field) across the 68 historical samples.

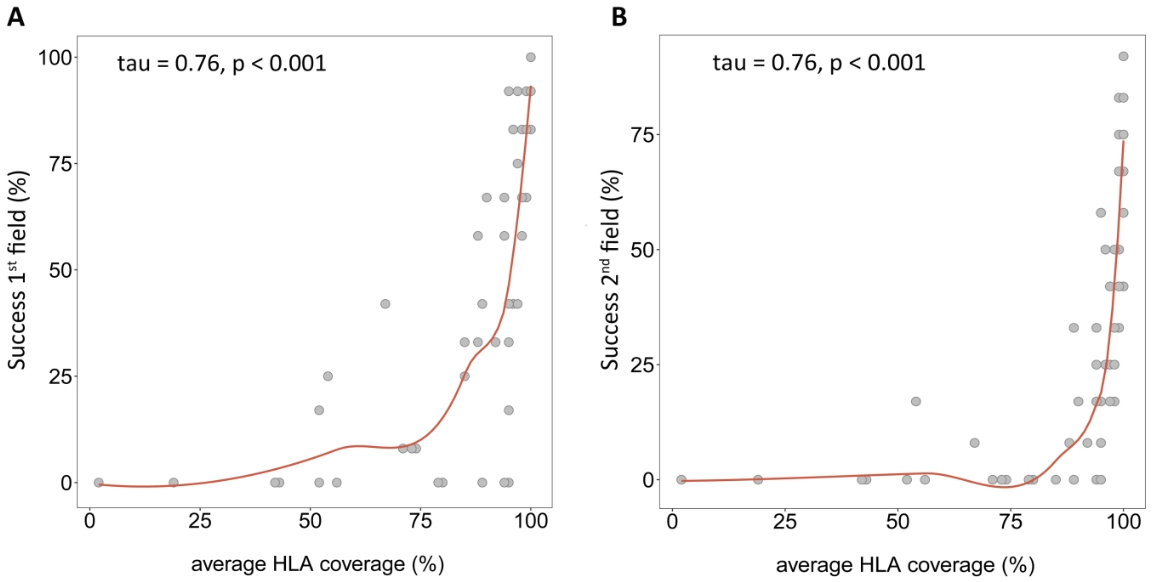

Figure S4. Correlation between average read depth over the HLA genes and success rate at two different levels of resolutions (A: 1st field; B: 2nd field) across the 68 historical samples.

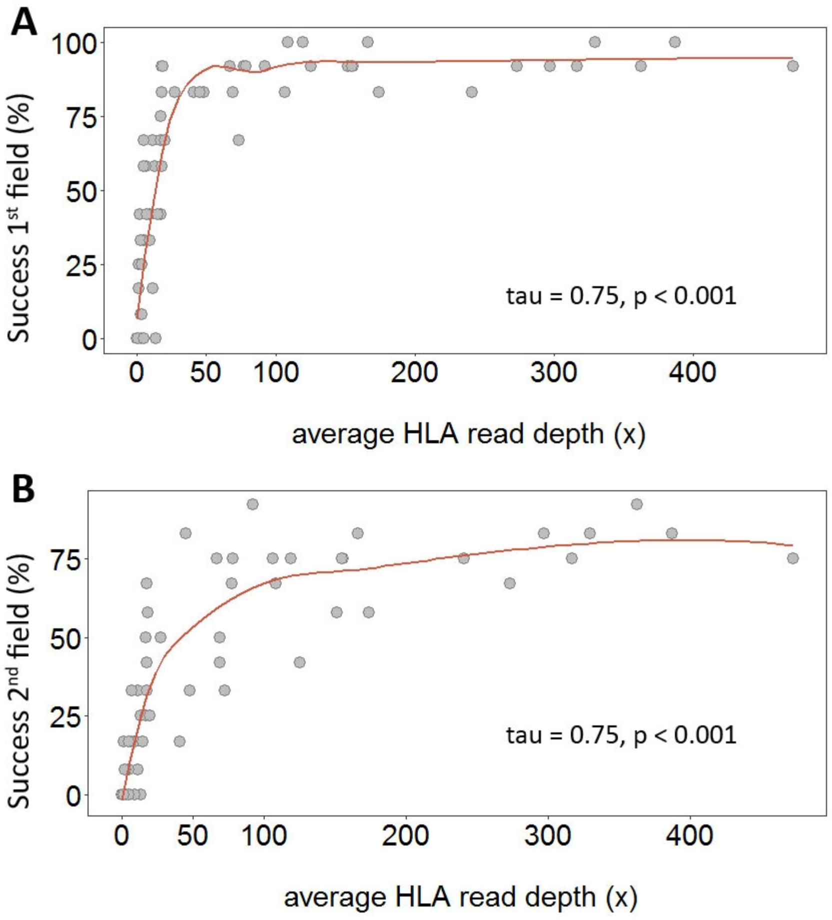

Figure S5. Correlation between average read depth over the HLA genes and success rate at two different levels of resolutions (A: 1st field; B: 2nd field) for the simulated aDNA samples.

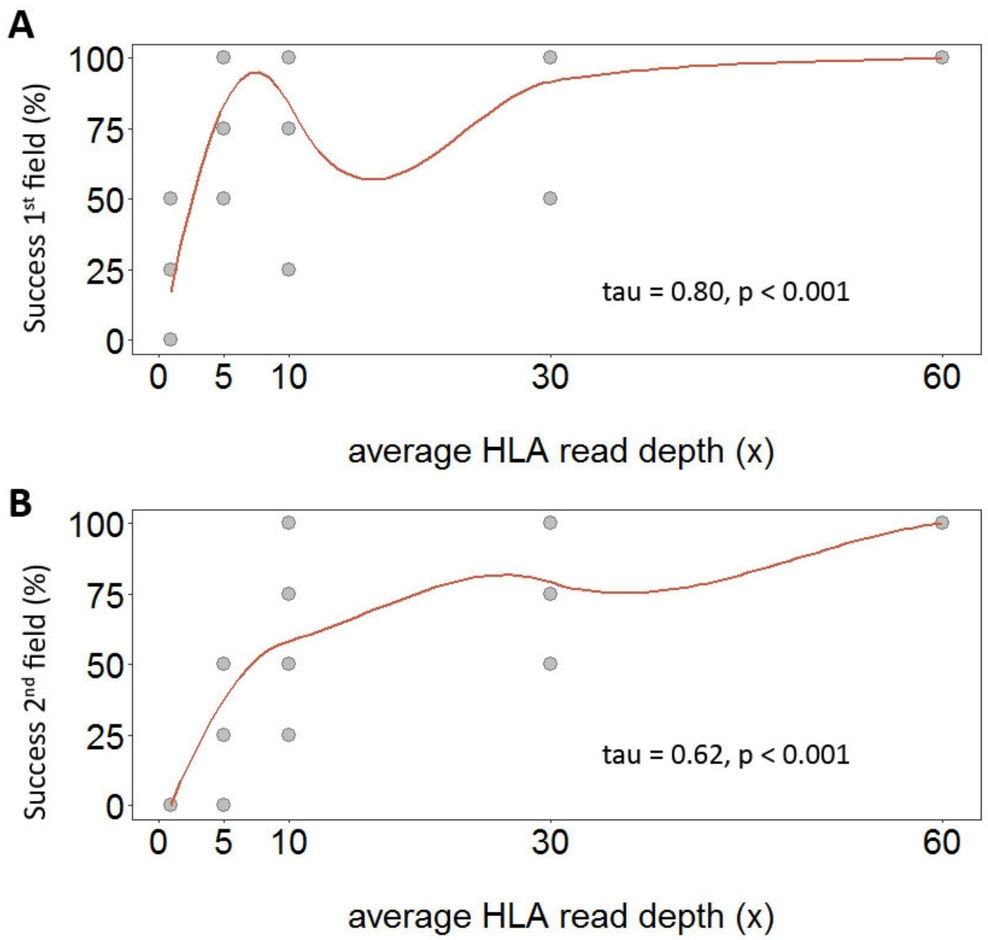

Figure S6. Screen-shot of pipeline output in the sequence alignment editor BioEdit. The picture shows the distinction between SNPs specific of each individual allele, typical aDNA base modifications or PCR/sequencing errors.

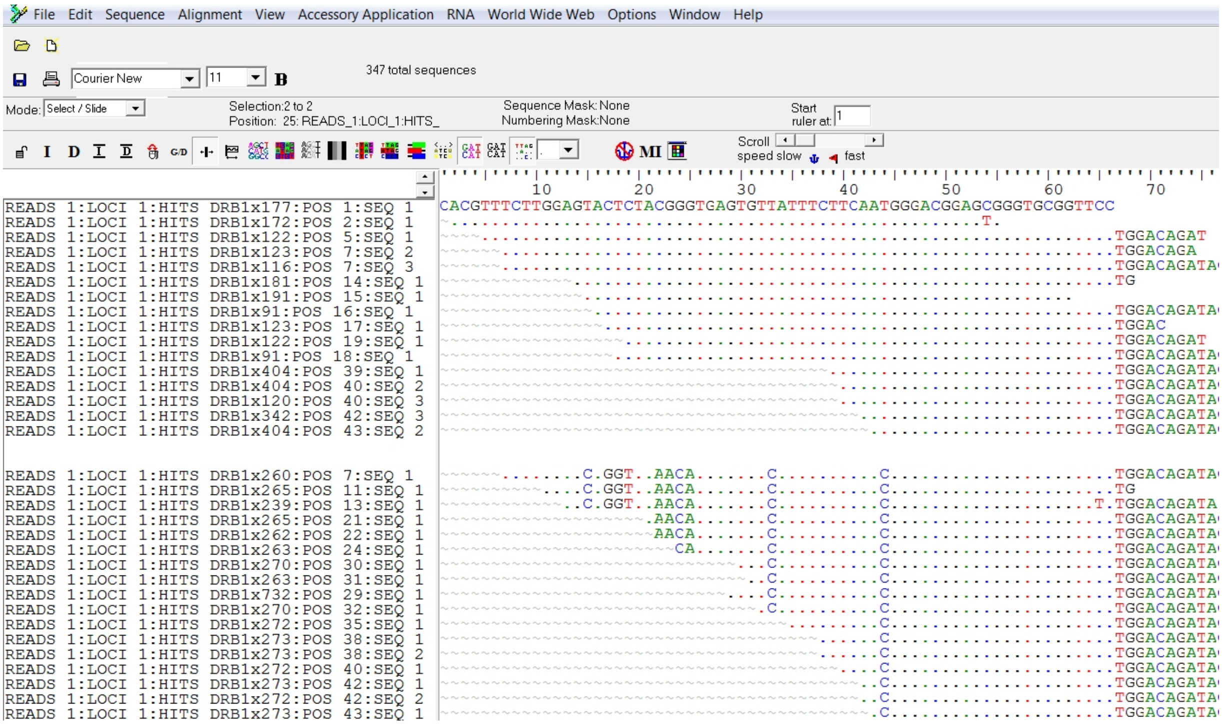

Note - the ID tag of each sequence in the FASTA file summarizes information from the sorting procedure performed by the Perl script. In the first domain, READS, is stored the observed number of reads of the exact same sequence fragment (representing PCR duplicates), while the actual sequence read is represented in the FASTA file only once. The second domain, LOCI, stores the number of HLA loci that this read is mapping to. The third domain, HITS, stores the name and allele number of all loci to which the read is mapping to. The fourth domain, POS, indicates the starting position of the reas along the locus. The fifth domain, SEQ, provides a unique number for reads starting from the same position.

Table S1. *H. sapiens* damage patterns of UDG datasets.

Individual	DMG 1st base 5' [%]	DMG 2nd base 5' [%]	DMG 3rd base 5' [%]	DMG 4th base 5' [%]	DMG 5th base 5' [%]	DMG 1st base 3' [%]	DMG 2nd base 3' [%]	DMG 3rd base 3' [%]	DMG 4th base 3' [%]	DMG 5th base 3' [%]
G022	1.49	0.74	0.51	0.43	0.36	1.49	0.86	0.63	0.51	0.45
G102	1.49	0.81	0.57	0.42	0.35	1.54	0.82	0.56	0.43	0.34
G104	1.57	0.85	0.59	0.44	0.38	1.51	0.82	0.58	0.44	0.39
G1042	0.97	0.82	0.54	0.42	0.43	0.92	0.84	0.54	0.49	0.41
G1044	1.25	0.7	0.52	0.37	0.35	1.17	0.68	0.46	0.37	0.33
G1049	1.69	1.05	0.82	0.64	0.56	1.55	0.97	0.77	0.62	0.57
G1065	1.39	0.79	0.61	0.48	0.4	1.45	0.79	0.61	0.48	0.4
G1083	1.18	0.59	0.51	0.36	0.32	1.09	0.58	0.45	0.35	0.33
G1137	1.28	0.73	0.48	0.38	0.32	1.35	0.76	0.49	0.4	0.34
G1149	1.68	0.91	0.69	0.56	0.44	1.74	0.91	0.69	0.56	0.46
G117	0.94	0.48	0.41	0.3	0.33	0.83	0.48	0.35	0.31	0.24
G118	0.91	0.59	0.45	0.37	0.31	0.85	0.51	0.42	0.34	0.26
G119	1.01	0.68	0.6	0.45	0.41	0.93	0.63	0.57	0.43	0.39
G120	1.4	0.87	0.68	0.47	0.43	1.29	0.82	0.63	0.45	0.42
G131	1.74	0.69	0.49	0.42	0.38	1.61	0.77	0.55	0.44	0.39
G140	1.32	0.83	0.64	0.43	0.4	1.16	0.74	0.55	0.45	0.37
G149	1.3	0.65	0.46	0.35	0.32	1.4	0.69	0.5	0.4	0.34
G154	1.73	0.91	0.65	0.53	0.44	1.65	0.85	0.63	0.51	0.42
G164	1.03	0.61	0.54	0.38	0.31	1.01	0.59	0.45	0.37	0.33
G165	1.09	0.87	0.66	0.5	0.44	1.02	0.8	0.62	0.46	0.43
G166	1.03	1.07	0.5	0.47	0.37	0.99	1.05	0.49	0.5	0.39
G189	1.97	0.67	0.43	0.33	0.27	1.57	0.74	0.47	0.36	0.3
G208	1.51	0.78	0.56	0.45	0.36	1.56	0.78	0.55	0.44	0.35
G21	1.83	1.04	0.79	0.64	0.51	1.9	1.04	0.79	0.63	0.5
G24	1.54	0.91	0.66	0.53	0.46	1.48	0.91	0.67	0.53	0.47
G255	1.17	0.67	0.45	0.34	0.32	0.99	0.62	0.44	0.33	0.29
G274	1.23	0.67	0.56	0.37	0.31	1.2	0.58	0.42	0.35	0.28
G28	0.85	0.92	0.46	0.42	0.32	0.88	0.88	0.45	0.41	0.32
G289	1.28	0.66	0.42	0.32	0.3	1.17	0.7	0.44	0.39	0.36
G300	1.59	0.74	0.58	0.47	0.38	1.63	0.76	0.58	0.47	0.37
G314	1.56	0.82	0.59	0.48	0.39	1.6	0.83	0.6	0.48	0.38
G33	1.6	0.9	0.67	0.54	0.43	1.64	0.91	0.67	0.53	0.42
G34	1.76	0.91	0.61	0.49	0.4	1.79	0.91	0.6	0.48	0.39
G348	1.21	0.75	0.5	0.41	0.34	1.15	0.69	0.45	0.41	0.35
G393	1.48	0.8	0.54	0.41	0.33	1.51	0.8	0.54	0.41	0.32
G397	1.13	0.7	0.5	0.35	0.33	1.08	0.57	0.46	0.34	0.31
G404	2.57	1.18	0.78	0.62	0.51	2.63	1.2	0.8	0.64	0.52
G417	1.11	0.67	0.41	0.33	0.27	1.1	0.66	0.41	0.33	0.28

G427	1.84	0.86	0.59	0.44	0.37	1.85	0.9	0.58	0.46	0.37
G43	1.32	0.69	0.54	0.43	0.36	1.35	0.7	0.54	0.43	0.36
G472	1.37	0.68	0.51	0.39	0.33	1.41	0.72	0.54	0.42	0.36
G48	1.07	0.71	0.52	0.41	0.35	1.07	0.69	0.5	0.4	0.35
G507	2.06	0.93	0.61	0.49	0.39	2.12	0.98	0.63	0.51	0.41
G533	1.95	0.93	0.61	0.52	0.41	1.85	0.97	0.66	0.56	0.45
G658	0.9	0.55	0.36	0.31	0.27	0.97	0.58	0.4	0.32	0.28
G669	0.92	0.73	0.45	0.45	0.37	0.96	0.74	0.44	0.44	0.37
G708	1.79	0.95	0.64	0.49	0.38	1.79	0.94	0.62	0.49	0.38
G712	1.53	1.01	0.72	0.6	0.52	1.59	1.01	0.74	0.61	0.52
G722	1.07	0.5	0.37	0.28	0.24	1.05	0.56	0.4	0.32	0.27
G730	1.97	1.1	0.83	0.67	0.53	1.99	1.1	0.83	0.66	0.52
G738	1.18	0.77	0.53	0.43	0.34	1.15	0.72	0.55	0.44	0.37
G749	1.3	0.66	0.5	0.4	0.34	1.44	0.75	0.53	0.44	0.37
G750	1.11	0.8	0.53	0.45	0.37	0.99	0.72	0.49	0.38	0.37
G860	1.64	1.03	0.78	0.65	0.59	1.5	0.97	0.75	0.62	0.58
G864	1.22	0.78	0.58	0.49	0.42	1.17	0.75	0.57	0.47	0.41
G870	1.1	0.63	0.49	0.35	0.31	1	0.58	0.43	0.31	0.3
G876	1.41	0.89	0.7	0.55	0.47	1.45	0.89	0.68	0.55	0.47
G896	0.8	0.55	0.37	0.3	0.26	0.75	0.55	0.37	0.29	0.26
G911	1.36	0.69	0.55	0.38	0.32	1.25	0.67	0.48	0.36	0.31
G912	1.18	0.67	0.62	0.4	0.36	1.04	0.63	0.56	0.37	0.35
G914	1.06	1.17	0.66	0.58	0.49	1.05	1.16	0.67	0.59	0.5
G936	1.09	0.7	0.65	0.48	0.42	0.97	0.64	0.61	0.45	0.42
G939	0.98	0.55	0.38	0.29	0.24	0.95	0.54	0.37	0.29	0.24
G942	1.18	0.66	0.49	0.36	0.3	1.12	0.64	0.44	0.34	0.3
G943	1.63	0.88	0.72	0.55	0.45	1.6	0.96	0.65	0.51	0.45
G951	1.38	0.72	0.61	0.41	0.37	1.21	0.65	0.57	0.42	0.37
G973	1.4	0.69	0.49	0.37	0.3	1.46	0.69	0.47	0.38	0.31
G978	1.25	0.67	0.48	0.38	0.33	1.35	0.7	0.5	0.41	0.35

Table S2. *H. sapiens* damage patterns of non UDG datasets.

Individual	DMG 1st base 5' [%]	DMG 2nd base 5' [%]	DMG 3rd base 5' [%]	DMG 4th base 5' [%]	DMG 5th base 5' [%]	DMG 1st base 3' [%]	DMG 2nd base 3' [%]	DMG 3rd base 3' [%]	DMG 4th base 3' [%]	DMG 5th base 3' [%]
G022	7.02	5.15	3.62	2.96	2.49	6.85	4.94	3.4	2.92	2.41
G102	17.77	10.8	8.14	6.93	5.57	17.67	10.65	8.01	6.81	5.48
G104	17.39	9.84	7.66	6.48	5.51	17.41	9.81	7.54	6.42	5.49
G1042	19.59	12.6	10.8	10.04	8.72	19.73	13.04	10.47	10.1	9.05
G1044	10.92	6.72	4.63	3.91	3.31	10.91	6.59	4.56	3.83	3.32
G1049	18.12	10.81	8.68	7.82	7.14	18.28	10.67	8.57	7.83	7.09
G1065	13.54	8.2	6.38	5.63	4.58	13.58	8.16	6.26	5.53	4.5
G1083	11.4	6.78	4.92	4.13	3.54	11.34	6.8	4.89	4.07	3.49
G1137	7.28	4.92	3.3	2.77	2.35	7.38	5.11	3.36	2.81	2.41
G1149	19.07	13.68	10.63	9.05	6.71	18.96	13.42	10.41	8.9	6.58
G117	15.8	9.32	7.44	6.24	5.1	16.16	9.41	7.25	5.9	5.01
G118	9.38	5.89	4.25	3.49	2.99	9.54	5.68	4.15	3.45	2.99
G119	12.26	7.69	6.57	6.21	5.41	11.97	7.43	6.35	6.03	5.22
G120	20.72	11.36	9.27	8.32	7.37	20.84	11.36	9.21	8.32	7.35
G131	8.03	5.44	3.97	3.45	2.89	8.17	5.41	3.81	3.39	2.85
G140	15.22	9.05	7.2	6.19	5.42	15.6	8.95	7.19	6.19	5.56
G149	8.42	4.92	3.23	2.59	2.13	8.71	5.13	3.3	2.67	2.12
G154	17.64	10.57	7.93	6.82	5.9	17.65	10.49	7.76	6.77	5.81
G164	10.89	6.77	5.3	4.5	3.96	11.13	6.9	5.25	4.57	3.99
G165	13.41	7.67	6.33	5.71	5.08	13.49	7.71	6.41	5.73	5.07
G166	16.92	10.17	7.98	6.81	6.05	16.97	9.88	7.66	6.9	6.02
G189	8.72	5.22	3.4	2.75	2.13	8.65	5.11	3.31	2.72	2.14
G208	9.82	5.36	3.83	3.28	2.85	9.72	5.26	3.71	3.24	2.8
G21	16.25	10.34	7.19	6.04	4.75	16.13	10.15	7.04	5.97	4.65
G24	7.41	1.64	1.08	0.95	0.72	7.32	1.64	1.08	0.94	0.72
G255	14.25	8.02	6.78	6.25	5.37	14.02	7.88	6.57	6.15	5.19
G274	14.43	8.38	6.25	5.4	4.67	14.6	8.43	6.34	5.38	4.64
G28	11.28	6.52	4.82	4.11	3.43	11.5	6.45	4.75	4.13	3.6
G289	13.34	7.94	6	5	4.36	13.17	7.85	5.94	5.12	4.46
G300	5.5	4.1	2.77	2.22	1.62	5.19	4	2.64	2.23	1.76
G314	15.56	9.86	7.07	5.97	4.66	15.49	9.72	6.96	5.83	4.56
G33	15.57	10.01	7.29	6.28	5.01	15.07	9.64	6.97	6.06	4.83
G34	21.94	15.14	11.53	9.74	7.38	21.89	14.84	11.22	9.51	7.16
G348	19.46	11.59	8.54	7.3	6.09	19.3	11.48	8.37	7.13	5.97
G393	19.61	11.9	8.52	6.91	5.34	19.65	11.78	8.35	6.72	5.2
G397	11.63	6.86	5.22	4.38	3.89	11.69	6.79	5.07	4.42	3.84
G404	14.08	9.09	3.53	2.53	0	12.05	1.27	3.41	2.02	2.35
G417	13.19	7.5	5.2	4.39	3.86	13.13	7.48	5.3	4.42	3.74

G427	10.09	7.33	4.73	3.74	2.89	10.18	7.18	4.64	3.71	2.96
G43	11.27	6.93	4.65	3.92	3.25	11.19	6.82	4.53	3.85	3.19
G472	13.86	9.13	5.98	4.88	3.77	13.84	9.02	5.91	4.84	3.84
G48	11.1	6.85	5.32	4.77	3.99	10.94	6.71	5.15	4.67	3.92
G507	9.23	6.56	4.67	3.93	3.05	9.37	6.56	4.65	3.87	3.08
G533	15.1	9.63	6.46	5.27	4.14	15.04	9.5	6.4	5.28	4.29
G658	5.32	4.1	2.79	2.31	1.78	5.25	4.02	2.78	2.25	1.89
G669	14.49	10.38	7.32	6.79	5.3	14.51	10.27	7.17	6.7	5.21
G708	20.9	13.04	9.87	8.26	6.5	20.63	12.84	9.65	8.11	6.33
G712	17.89	13.29	11.49	10.46	8.77	17.77	13.16	11.28	10.44	8.66
G722	4.62	3.26	2.15	1.74	1.51	4.71	3.35	2.19	1.81	1.49
G730	8.25	2.48	1.77	1.54	1.21	8.11	2.45	1.73	1.56	1.19
G738	10.26	7.22	5.8	5.08	3.97	9.9	6.93	5.41	4.94	3.94
G749	9.34	6.3	3.99	3.33	2.63	9.24	6.11	3.88	3.28	2.65
G750	13.8	8.73	7.05	6.49	5.57	13.7	8.66	6.89	6.47	5.47
G860	13.02	7.58	6.3	5.94	5.52	13.01	7.56	6.24	5.92	5.45
G864	11.44	6.91	5.53	4.95	4.29	11.2	6.73	5.34	4.85	4.22
G870	12.15	6.91	5.26	4.58	3.73	11.83	6.64	4.99	4.36	3.64
G876	10.12	5.62	4.39	4.01	3.12	10	5.47	4.28	3.91	3.03
G896	8.35	4.58	3.36	2.91	2.55	8.23	4.6	3.36	2.92	2.55
G911	11.46	5.93	4.19	3.41	2.86	11.53	5.92	4.2	3.46	2.9
G912	13.66	7.43	6.1	5.34	4.68	13.87	7.53	6.12	5.37	4.71
G914	17.21	10.57	8.97	8.72	8.06	17.14	10.5	8.93	8.81	7.95
G936	14.68	8.3	7	6.24	5.71	14.6	8.3	6.93	6.28	5.67
G939	9.32	5.25	3.68	3.13	2.47	9.19	5.25	3.62	3.1	2.46
G942	11.79	6.37	4.31	3.54	2.98	11.68	6.34	4.31	3.52	3.01
G943	14.24	9.69	7.34	6.82	5.33	14.35	9.23	7.37	6.64	5.16
G951	12.33	7.1	5.74	4.92	4.42	12.43	7.19	5.75	5.06	4.41
G973	15.61	9.37	6.51	5.32	4.34	15.32	9.15	6.32	5.2	4.23
G978	6.14	4.12	2.86	2.24	1.78	5.72	4.15	2.74	2.26	1.83

Table S3. Median (range) read lengths of UDG datasets.

Individual	read length
G022	77 (57-101)
G102	54 (42-72)
G104	51 (41-66)
G1042	47 (39-61)
G1044	75 (61-76)
G1049	61 (47-76)
G1065	62 (45-76)
G1083	67 (48-76)
G1137	84 (61-109)
G1149	68 (48-76)
G117	68 (50-76)
G118	74 (54-76)
G119	58 (46-75)
G120	55 (44-71)
G131	76 (55-101)
G140	64 (48-76)
G149	87 (66-107)
G154	68 (49-76)
G164	67 (49-76)
G165	62 (48-76)
G166	76 (56-77)
G189	74 (55-94)
G208	60 (45-76)
G21	48 (38-62)
G24	55 (44-69)
G255	73 (54-76)
G274	71 (51-76)
G28	76 (59-78)
G289	85 (61-113)
G300	59 (44-76)
G314	60 (45-76)
G33	58 (43-76)
G34	54 (41-74)
G348	70 (49-76)
G393	49 (38-65)
G397	75 (60-76)
G404	85 (61-107)
G417	75 (52-77)
G427	91 (64-109)

G43	62 (46-76)
G472	74 (56-96)
G48	55 (44-71)
G507	80 (58-102)
G533	93 (68-123)
G658	78 (59-107)
G669	57 (42-75)
G708	55 (43-71)
G712	59 (44-76)
G722	82 (61-112)
G730	50 (39-65)
G738	64 (48-76)
G749	97 (67-125)
G750	53 (41-71)
G860	63 (49-76)
G864	58 (43-75)
G870	66 (48-76)
G876	60 (45-76)
G896	76 (66-80)
G911	76 (62-78)
G912	73 (53-76)
G914	61 (48-76)
G936	58 (46-75)
G939	74 (52-76)
G942	75 (62-76)
G943	55 (43-76)
G951	61 (48-76)
G973	66 (48-76)
G978	101 (73-115)

Table S4. Comparison of sequence data before and after HLA enrichment experiments reported for a subset of 62 historical samples.

sample	sequence data	total reads	reads aligning to hg19	endogenous (%)	reads aligning to HLA genes	% of reads aligning to HLA genes	HLA fold-enrichments
G022	original	8599437	56925	1	0	0.000	493.00
	HLA enriched	10851598	278504	3	493	0.005	
G102	original	29473512	7055498	24	32	0.000	2429.75
	HLA enriched	48872766	16524082	34	77752	0.159	
G104	original	16762492	2273849	14	14	0.000	1159.21
	HLA enriched	18296360	4331589	24	16229	0.089	
G1044	original	11939954	1474786	12	4	0.000	11717.50
	HLA enriched	30191012	12147837	40	46870	0.155	
G1049	original	19490520	4863184	25	8	0.000	9708.25
	HLA enriched	42578983	18618541	44	77666	0.182	
G1065	original	36441574	23593147	65	109	0.000	2064.34
	HLA enriched	65461148	44052176	67	225013	0.344	
G1083	original	408892110	16764367	4	57	0.000	163.00
	HLA enriched	18962338	2923148	15	9291	0.049	
G1137	original	174340757	10896459	6	49	0.000	244.51
	HLA enriched	56265338	12331989	22	11981	0.021	
G1149	original	414092343	49259685	12	137	0.000	1341.17
	HLA enriched	85890554	60138238	70	183740	0.214	
G117	original	10235336	147869	1	0	0.000	2836.00
	HLA enriched	15701994	976619	6	2836	0.018	
G118	original	12907976	219935	2	0	0.000	4521.00
	HLA enriched	29259916	906802	3	4521	0.015	
G119	original	14841003	2686014	18	9	0.000	13596.11
	HLA enriched	117183091	32594583	28	122365	0.104	
G120	original	18132253	5381710	30	23	0.000	5577.00
	HLA enriched	63535664	26550293	42	128271	0.202	
G131	original	17278424	176996	1	0	0.000	359.00
	HLA enriched	9515007	253355	3	359	0.004	
G140	original	7628920	106989	1	1	0.000	3484.00
	HLA enriched	14312395	596777	4	3484	0.024	
G149	original	9196823	443414	5	5	0.000	1157.80
	HLA enriched	36304032	7004939	19	5789	0.016	
G154	original	240299649	91470953	38	378	0.000	400.35
	HLA enriched	62627578	34744640	55	151333	0.242	
G164	original	16248305	624401	4	0	0.000	3084.00
	HLA enriched	28650945	1201882	4	3084	0.011	

G165	original	19328959	4569589	24	19	0.000	7032.21
	HLA enriched	45755967	22552165	49	133612	0.292	
G189	original	51568721	5879989	11	8452	0.016	2.97
	HLA enriched	37770246	13518812	36	25095	0.066	
G208	original	286129178	70862102	25	259	0.000	616.76
	HLA enriched	56621036	36900228	65	159741	0.282	
G21	original	23972210	12964863	54	48	0.000	2511.29
	HLA enriched	49630066	35396253	71	120542	0.243	
G24	original	30498430	8071032	26	34	0.000	2080.82
	HLA enriched	57474432	15081426	26	70748	0.123	
G255	original	13384857	340867	3	2	0.000	5851.00
	HLA enriched	28720784	3716194	13	11702	0.041	
G274	original	14859656	393268	3	1	0.000	4427.00
	HLA enriched	15030111	1112193	7	4427	0.029	
G289	original	16927524	160918	1	1	0.000	386.00
	HLA enriched	9978233	244945	2	386	0.004	
G300	original	15439492	10677785	69	35	0.000	1512.71
	HLA enriched	28579922	15262686	53	52945	0.185	
G314	original	31398694	14536475	46	40	0.000	2290.55
	HLA enriched	39074393	24212018	62	91622	0.234	
G33	original	246906567	13095876	5	47	0.000	956.36
	HLA enriched	37557603	14434462	38	44949	0.120	
G34	original	382282413	137542840	36	519	0.000	163.24
	HLA enriched	48638831	19843954	41	84721	0.174	
G348	original	16754756	2295115	14	12	0.000	1044.08
	HLA enriched	18109228	4175796	23	12529	0.069	
G393	original	35355816	15875665	45	61	0.000	2028.85
	HLA enriched	64103915	32163347	50	123760	0.193	
G397	original	12425806	590819	5	2	0.000	12547.50
	HLA enriched	25663966	5379042	21	25095	0.098	
G404	original	219936423	14371069	7	49	0.000	169.57
	HLA enriched	40563956	11014098	27	8309	0.020	
G427	original	245763525	9384556	4	35	0.000	349.29
	HLA enriched	171289835	13167034	8	12225	0.007	
G43	original	35150927	17599123	50	57	0.000	3581.75
	HLA enriched	77091157	51187499	66	204160	0.265	
G472	original	243418738	27042316	11	132	0.000	183.96
	HLA enriched	64528676	23686463	37	24283	0.038	
G48	original	21187364	9869247	47	26	0.000	2470.00
	HLA enriched	25368973	13697638	54	64220	0.253	

G507	original	237551028	97709253	41	409	0.000	117.97
	HLA enriched	86310325	58291258	68	48248	0.056	
G533	original	28125125	2864347	10	13	0.000	1246.54
	HLA enriched	41730888	10265836	25	16205	0.039	
G658	original	548165791	46394838	8	162	0.000	37.37
	HLA enriched	57576755	7410760	13	6054	0.011	
G669	original	40030680	18633086	47	66	0.000	2642.26
	HLA enriched	83187634	56356467	68	174389	0.210	
G708	original	29202942	6861354	23	27	0.000	2942.22
	HLA enriched	49400523	17902389	36	79440	0.161	
G712	original	29076810	7897978	27	37	0.000	1314.62
	HLA enriched	25073439	10471184	42	48641	0.194	
G722	original	412635307	57950390	14	156	0.000	40.13
	HLA enriched	35231822	8704397	25	6261	0.018	
G730	original	30979029	8017931	26	39	0.000	2122.05
	HLA enriched	32166123	17368044	54	82760	0.257	
G738	original	36353460	1437603	4	3	0.000	3812.67
	HLA enriched	55485529	2720910	5	11438	0.021	
G749	original	22577271	5329614	24	12	0.000	1096.17
	HLA enriched	40918094	16020855	39	13154	0.032	
G750	original	16027731	1129349	7	7	0.000	754.71
	HLA enriched	5496339	1016297	18	5283	0.096	
G860	original	20727017	15675250	76	99	0.000	1752.87
	HLA enriched	53408873	36828601	69	173534	0.325	
G864	original	18458498	12683368	69	54	0.000	1411.33
	HLA enriched	19567241	13942742	71	76212	0.389	
G870	original	17782756	1009246	6	7	0.000	4071.14
	HLA enriched	23287681	5015827	22	28498	0.122	
G876	original	30275792	14274672	47	69	0.000	1439.75
	HLA enriched	41966479	22663531	54	99343	0.237	
G911	original	12122972	3271201	27	12	0.000	4809.08
	HLA enriched	26377084	14688225	56	57709	0.219	
G912	original	16458021	1682533	10	3	0.000	8077.33
	HLA enriched	17875627	5299164	30	24232	0.136	
G936	original	18568653	3038829	16	21	0.000	9137.67
	HLA enriched	84300687	74490774	88	191891	0.228	
G939	original	14134206	4505953	32	11	0.000	8090.55
	HLA enriched	47798424	26595569	56	88996	0.186	
G942	original	18190020	2211172	12	7	0.000	11819.43
	HLA enriched	54209829	22938407	42	82736	0.153	

G943	original	170193606	2110175	1	5	0.000	484.20
	HLA enriched	19445661	418647	2	2421	0.012	
G951	original	16358004	1243961	8	3	0.000	3349.00
	HLA enriched	24554661	3164285	13	10047	0.041	
G973	original	15462662	5926620	38	17	0.000	3889.47
	HLA enriched	39044885	22012088	56	66121	0.169	
G978	original	221493132	3906293	2	17	0.000	243.35
	HLA enriched	109771423	5684949	5	4137	0.004	

Note - Endogenous DNA content and percentage of reads aligning to HLA genes calculated from original UDG shotgun libraries and HLA-enriched UDG libraries are reported for each individual sample, for a total of 62 ancient samples. Fold-enrichment was obtained by dividing the number of reads mapping to the HLA reference obtained from enriched HLA libraries by the number of reads mapping to the HLA reference calculated from pre-capture shotgun libraries; when the denominator was 0 the number of on-target HLA reads from enriched libraries has been assigned.

Table S5. Coverage and read depth at each locus and averaged across the 6 investigated genes before and after HLA enrichment experiments reported for a subset of 62 historical samples.

sample	sequence data	HLA-A		HLA-B		HLA-C		HLA-DRB1		HLA-DQB1		HLA-DPB1		average HLA cov [%]	average HLA read depth
		cov [%]	read depth	cov [%]	read depth	cov [%]	read depth	cov [%]	read depth	cov [%]	read depth	cov [%]	read depth		
G022	original	0	0.00	0	0.0	0	0.00	0	0.00	0	0.00	0	0.00	0	0.00
	HLA enriched	18	0.23	0	0.1	23	0.36	14	0.33	39	0.49	0	0.00	19	0.31
G102	original	8	0.22	6	0.2	0	0.17	0	0.00	14	0.14	21	0.21	6	0.14
	HLA enriched	98	53.42	99	59.4	100	52.29	99	45.17	94	29.88	98	27.76	98	48.03
G104	shotgun	23	0.44	12	0.25	0	0.09	0	0.00	0	0.00	0	0.00	7	0.16
	HLA enriched	97	17.96	98	14.60	97	14.34	85	5.54	98	6.61	96	6.68	95	11.81
G1044	original	16	0.19	0	0.0	0	0.03	0	0.00	0	0.00	0	0.00	3	0.05
	HLA enriched	98	28.98	97	15.0	96	16.21	94	11.72	92	14.61	94	12.04	96	17.30
G1049	original	0	0.08	0	0.1	0	0.02	22	0.29	0	0.00	14	0.14	4	0.10
	HLA enriched	100	127.86	100	133.2	100	119.43	99	77.36	100	73.55	98	61.28	100	106.29
G1065	original	77	1.62	52	1.2	38	0.77	23	0.64	62	0.71	15	0.26	50	0.98
	HLA enriched	100	606.03	100	537.8	100	504.98	100	349.45	100	364.80	98	311.15	100	472.62
G1083	original	33	0.33	27	0.5	0	0.24	0	0.00	0	0.00	0	0.00	12	0.22
	HLA enriched	76	4.75	91	5.7	82	5.50	99	10.29	74	2.15	12	0.25	85	5.67
G1137	original	15	0.31	23	0.4	6	0.28	36	0.46	32	0.31	27	0.33	22	0.36
	HLA enriched	72	1.37	73	4.2	70	3.19	84	2.67	56	1.36	60	0.94	71	2.56
G1149	original	75	1.56	38	0.8	43	0.88	52	0.78	70	1.39	53	0.59	56	1.08
	HLA enriched	100	462.14	100	389.4	100	388.26	100	289.73	100	284.29	98	250.32	100	362.77
G117	original	0	0.00	0	0.0	0	0.00	0	0.00	0	0.00	0	0.00	0	0.00
	HLA enriched	61	1.99	63	1.3	41	1.20	36	1.15	57	1.40	55	0.95	52	1.41
G118	original	0	0.00	0	0.0	0	0.00	0	0.00	0	0.00	0	0.00	0	0.00
	HLA enriched	80	4.53	88	4.4	94	4.06	75	1.21	65	1.38	66	1.41	80	3.11
G119	original	0	0.09	15	0.3	0	0.14	40	0.10	10	0.10	0	0.00	13	0.14
	HLA enriched	100	201.52	100	201.4	100	184.83	100	116.40	100	127.56	98	95.18	100	166.34
G120	original	0	0.08	3	0.2	3	0.17	20	0.27	0	0.00	0	0.00	5	0.14
	HLA enriched	100	91.42	100	85.6	100	73.98	97	67.85	100	46.67	95	33.21	99	73.11
G131	original	0	0.00	0	0.0	0	0.00	0	0.00	0	0.00	0	0.00	0	0.00
	HLA enriched	47	1.11	40	0.8	44	0.92	21	0.70	62	1.32	28	0.39	43	0.97
G140	original	0	0.00	0	0.0	0	0.00	0	0.00	0	0.00	0	0.00	0	0.00
	HLA enriched	98	18.64	97	19.2	97	17.04	95	11.12	89	3.26	93	11.84	95	13.84
G149	original	0	0.03	0	0.0	0	0.00	0	0.00	12	0.12	36	0.36	2	0.03
	HLA enriched	34	1.24	48	0.9	37	1.16	84	2.10	77	1.31	82	1.97	56	1.35
G154	original	82	2.60	84	2.6	82	1.68	48	0.92	77	2.04	41	0.92	75	1.97
	HLA enriched	98	56.65	99	46.4	99	44.51	96	41.66	98	17.26	97	15.09	98	41.29

G164	original	0	0.00	0	0.0	0	0.00	0	0.00	0	0.00	0	0.00	0	0.00
	HLA enriched	96	7.12	95	6.2	91	4.47	96	4.76	91	2.77	61	1.25	94	5.07
G165	original	22	0.42	8	0.1	0	0.09	0	0.00	0	0.00	19	0.19	6	0.12
	HLA enriched	100	93.40	100	83.8	100	84.92	99	49.04	99	35.77	98	41.78	99	69.38
G189	original	59	1.07	69	1.6	61	1.27	63	1.20	87	1.27	0	0.04	68	1.29
	HLA enriched	96	10.85	94	13.9	96	11.31	96	8.93	93	3.42	88	6.79	95	9.68
G208	original	74	1.61	74	1.8	60	2.29	63	1.09	94	1.52	57	0.88	73	1.66
	HLA enriched	100	140.98	100	138.9	100	142.55	100	103.31	100	100.74	98	83.59	100	125.30
G21	original	28	0.45	6	0.2	18	0.27	0	0.17	50	0.50	30	0.36	20	0.31
	HLA enriched	100	188.99	100	172.5	100	159.81	100	118.05	100	118.89	97	92.83	100	151.65
G24	original	17	0.35	6	0.1	13	0.22	21	0.23	0	0.02	0	0.02	11	0.18
	HLA enriched	100	225.47	100	222.0	100	199.88	100	128.56	100	95.55	98	100.75	100	174.29
G255	original	8	0.11	0	0.0	0	0.03	0	0.00	0	0.00	0	0.00	2	0.03
	HLA enriched	77	5.06	78	3.7	82	5.00	54	2.00	78	2.73	92	3.75	74	3.69
G274	original	8	0.09	0	0.0	0	0.00	0	0.00	0	0.00	0	0.00	2	0.02
	HLA enriched	91	6.21	70	3.2	60	2.58	86	2.29	87	3.52	56	2.32	79	3.56
G289	original	12	0.12	0	0.0	0	0.00	0	0.00	0	0.00	0	0.00	2	0.02
	HLA enriched	45	0.77	56	0.8	51	0.62	0	0.02	59	0.64	53	0.99	42	0.58
G300	original	28	0.41	17	0.4	19	0.39	0	0.18	0	0.00	24	0.24	13	0.27
	HLA enriched	100	306.00	100	262.5	100	240.71	100	215.92	100	181.40	98	148.22	100	241.30
G314	original	8	0.17	0	0.2	15	0.41	0	0.21	26	0.26	23	0.23	10	0.24
	HLA enriched	100	142.28	100	130.8	100	134.82	100	101.30	100	88.67	98	78.65	100	119.58
G33	original	15	0.22	19	0.3	0	0.09	0	0.00	0	0.00	0	0.00	7	0.12
	HLA enriched	99	87.80	99	81.4	100	66.98	97	60.40	100	48.24	97	41.41	99	68.95
G34	original	96	4.40	96	3.7	91	3.00	76	3.60	99	5.22	93	3.39	91	3.98
	HLA enriched	100	124.39	100	119.4	100	105.55	100	118.37	100	74.45	98	61.76	100	108.43
G348	original	16	0.20	0	0.0	0	0.08	15	0.22	0	0.00	0	0.07	6	0.11
	HLA enriched	96	11.29	96	11.4	95	11.52	85	8.27	96	13.45	85	12.16	94	11.19
G393	original	45	0.63	25	0.5	23	0.47	0	0.13	41	0.53	0	0.06	27	0.45
	HLA enriched	100	113.52	100	106.0	100	105.14	100	71.93	100	65.56	98	54.91	100	92.43
G397	original	0	0.00	0	0.0	0	0.00	0	0.00	0	0.00	0	0.00	0	0.00
	HLA enriched	93	15.40	98	16.8	98	14.10	97	14.86	83	5.49	84	3.99	94	13.32
G404	original	21	0.33	22	0.4	4	0.20	0	0.00	39	0.41	57	0.57	17	0.26
	HLA enriched	96	7.35	95	7.0	88	7.24	95	4.44	97	5.12	78	5.47	94	6.24
G427	original	42	0.71	0	0.1	23	0.35	36	0.45	31	0.31	0	0.09	26	0.39
	HLA enriched	89	3.75	86	3.6	91	4.45	80	5.08	78	2.33	81	2.25	85	3.84
G43	original	20	0.42	28	0.5	23	0.45	0	0.12	42	0.44	0	0.05	23	0.38
	HLA enriched	100	497.30	100	451.7	100	389.18	100	322.74	100	277.25	98	241.24	100	387.63
G472	original	50	0.96	67	1.0	43	0.88	50	0.76	22	0.30	57	1.09	46	0.78
	HLA enriched	92	7.64	96	5.7	96	6.37	97	3.84	80	1.29	70	3.13	92	4.98

G48	original	12	0.28	0	0.2	0	0.27	0	0.00	14	0.14	0	0.00	5	0.17
	HLA enriched	99	55.35	100	58.8	99	51.10	98	36.73	97	24.19	97	19.97	99	45.24
G507	original	88	3.21	86	2.8	73	2.19	73	3.54	76	1.18	72	1.70	79	2.58
	HLA enriched	97	17.43	98	21.7	98	18.45	99	20.38	95	9.15	94	5.19	97	17.42
G533	original	6	0.12	17	0.3	0	0.04	46	0.67	0	0.00	0	0.00	14	0.22
	HLA enriched	78	6.23	95	6.3	89	4.79	90	3.62	89	3.84	65	2.09	88	4.95
G658	original	76	1.58	67	1.2	51	1.02	81	2.70	67	0.99	65	1.09	68	1.50
	HLA enriched	98	6.18	92	4.2	80	3.56	89	7.84	92	3.77	92	7.01	90	5.11
G669	original	17	0.24	34	0.5	24	0.37	20	0.45	53	0.71	22	0.32	30	0.46
	HLA enriched	100	396.57	100	311.3	100	309.92	100	252.61	100	216.81	98	192.93	100	297.44
G708	original	12	0.27	0	0.1	0	0.17	0	0.00	0	0.00	0	0.00	2	0.12
	HLA enriched	100	77.52	99	79.9	100	72.18	99	56.53	100	49.37	97	37.03	100	67.09
G712	original	20	0.39	0	0.2	8	0.27	0	0.23	0	0.00	0	0.08	6	0.21
	HLA enriched	100	211.86	100	161.1	100	177.42	100	132.34	100	96.72	97	109.71	100	155.89
G722	original	63	1.75	53	1.4	54	0.94	42	0.62	99	1.78	31	0.31	62	1.31
	HLA enriched	77	3.85	92	3.9	87	2.61	85	2.66	99	2.12	79	2.43	88	3.02
G730	original	11	0.22	14	0.3	9	0.19	0	0.09	22	0.22	18	0.18	11	0.20
	HLA enriched	100	100.89	100	91.5	100	86.16	100	54.41	99	53.78	97	43.96	99	77.35
G738	original	2	0.07	0	0.0	0	0.00	0	0.00	0	0.00	0	0.00	0	0.01
	HLA enriched	99	13.04	97	11.6	93	10.59	91	7.48	96	5.35	86	7.73	95	9.61
G749	original	0	0.00	0	0.0	47	0.48	16	0.16	17	0.17	15	0.15	16	0.16
	HLA enriched	59	1.76	73	1.4	77	2.90	69	3.87	59	0.66	84	4.53	67	2.12
G750	original	0	0.07	0	0.0	11	0.15	0	0.12	0	0.00	0	0.08	2	0.07
	HLA enriched	97	22.68	98	20.1	99	18.07	97	15.82	97	12.68	96	9.88	98	17.87
G860	original	54	1.31	52	1.1	55	0.93	52	0.87	65	1.13	81	1.01	56	1.06
	HLA enriched	100	418.27	100	386.6	100	380.91	100	281.45	100	183.29	98	200.72	100	330.11
G864	original	11	0.26	19	0.4	23	0.44	21	0.34	0	0.00	51	0.97	15	0.28
	HLA enriched	100	336.17	100	310.7	100	293.96	100	257.25	100	171.12	98	162.23	100	273.85
G870	original	5	0.05	0	0.0	0	0.03	16	0.20	0	0.00	16	0.16	4	0.06
	HLA enriched	99	19.93	97	15.3	97	16.51	96	12.80	97	9.56	94	9.19	97	14.83
G876	original	26	0.43	39	0.7	41	0.68	0	0.00	50	0.71	0	0.00	31	0.50
	HLA enriched	100	407.98	100	352.7	100	333.26	100	277.28	100	213.86	98	189.30	100	317.02
G911	original	7	0.14	0	0.1	0	0.08	0	0.13	0	0.00	20	0.20	1	0.09
	HLA enriched	95	32.83	98	26.6	98	26.16	99	31.71	90	20.35	89	15.00	96	27.53
G912	original	0	0.00	0	0.0	0	0.04	0	0.00	0	0.00	0	0.00	0	0.02
	HLA enriched	96	20.79	97	20.7	97	25.32	98	11.52	96	9.97	96	13.07	97	17.67
G936	original	0	0.12	11	0.3	0	0.13	20	0.20	0	0.00	12	0.11	6	0.15
	HLA enriched	100	115.71	100	100.8	100	91.91	100	45.62	100	39.41	98	30.54	100	78.69
G939	original	0	0.04	0	0.0	0	0.02	0	0.07	40	0.40	0	0.00	8	0.11
	HLA enriched	96	19.34	98	24.8	98	21.44	99	23.31	98	10.52	96	16.30	98	19.88
G942	original	0	0.00	14	0.2	9	0.12	0	0.05	0	0.00	24	0.30	5	0.07

	HLA enriched	98	27.77	95	22.4	96	20.27	96	17.73	91	4.31	97	13.76	95	18.49
G943	original	0	0.00	0	0.0	0	0.00	0	0.00	0	0.00	0	0.00	0	0.00
	HLA enriched	96	6.53	98	5.7	94	5.04	76	4.36	81	2.34	91	2.28	89	4.79
G951	original	6	0.06	0	0.0	0	0.00	0	0.00	0	0.00	0	0.00	1	0.01
	HLA enriched	99	20.26	97	17.9	99	21.83	99	16.36	99	14.39	93	14.04	99	18.14
G973	original	0	0.04	0	0.1	7	0.15	11	0.19	12	0.12	0	0.06	6	0.11
	HLA enriched	100	177.29	100	157.3	100	164.78	100	162.15	100	112.80	98	92.54	100	154.86
G978	original	0	0.06	0	0.1	19	0.25	0	0.00	37	0.37	29	0.29	11	0.15
	HLA enriched	35	0.51	47	1.6	50	1.03	53	0.81	75	2.45	79	1.35	52	1.28

Table S6. Median of coverage and read depth at each locus before and after HLA enrichment experiments reported for a subset of 62 historical samples.

	Coverage (%)						Read Depth (x)					
	original			HLA enriched			original			HLA enriched		
	Min	Max	Median (95% CI)	Min	Max	Median (95% CI)	Min	Max	Median (95% CI)	Min	Max	Median (95% CI)
HLA A	0	96	11 (7-17)	7	100	98 (96-100)	0	4.4	0.19 (0.09-0.31)	0.18	606.03	21.74 (11.29-87.80)
HLA B	0	96	1 (0-15)	0	100	98 (96-100)	0	3.67	0.16 (0.06-0.28)	0.13	537.84	21.21 (11.58-81.35)
HLA C	0	91	1 (0-13)	2	100	98 (96-100)	0	3	0.17 (0.08-0.27)	0.2	504.98	20.85 (11.31-72.18)
HLA DRB1	0	81	0 (0-16)	0	100	97 (96-99)	0	3.6	0.12 (0.00-0.20)	0.02	349.45	17.04 (8.93-49.04)
HLA DQB1	0	99	0 (0-22)	0	100	97 (92-99)	0	5.22	0.00 (0.00-0.22)	0.05	364.8	13.06 (4.31-39.41)
HLA DPB1	0	93	(0-18)	0	98	95(89-97)	0	3.39	0.08 (0.00-0.19)	0	311.15	13.42 (6.79-33.21)

Table S7. Allele calls at HLA class I and class II genes for the 68 historical samples (HLA class II data from Krause-Kyora et al. 2018).

Individual	A1	A2	B1	B2	C1	C2	DRB1 1	DRB1 2	DQB1 1	DQB1 2	DPB1 1	DPB1 2
G022	NA	NA	NA	NA	NA	NA	NA	NA	NA	NA	NA	NA
G102	A*02	NA	B*18	B*44	C*07	C*03	DRB1*04:03	DRB1*11	DQB1*03:01	DQB1*03:02	DPB1*04:02	NA
G104	NA	NA	NA	NA	NA	NA	DRB1*04	NA	NA	NA	DPB1*422:01	NA
G1042	NA	NA	NA	NA	NA	NA	NA	NA	NA	NA	NA	NA
G1044	A*03:01	NA	NA	NA	C*03	NA	NA	NA	DQB1*03:02	DQB1*06	DPB1*03:01	NA
G1049	A*03:01	A*03:01	B*07:02	B*07:02	C*07	NA	DRB1*14:01	DRB1*15:01	DQB1*05:03	DQB1*06:02	DPB1*452:01	NA
G1065	A*03:01	A*11:01	B*07:02	B*35:01	C*07	C*04	DRB1*01:01	DRB1*04:01	DQB1*03:01	DQB1*05:01	DPB1*04:01	NA
G1083	NA	NA	NA	NA	NA	NA	DRB1*01	DRB1*08	DQB1*04	DQB1*05	NA	NA
G1137	NA	NA	NA	NA	NA	NA	NA	NA	DQB1*02	NA	NA	NA
G1149	A*02:01	A*03:01	B*07:02	B*44:02	C*05:01	C*07:02	DRB1*04:01	DRB1*15:01	DQB1*03:01	DQB1*06:02	DPB1*04:01	NA
G117	NA	NA	NA	NA	NA	NA	NA	NA	NA	NA	NA	NA
G118	NA	NA	NA	NA	NA	NA	NA	NA	NA	NA	NA	NA
G119	A*24:02	A*24:02	B*07:02	B*07:02	C*07	C*07	DRB1*15:01	DRB1*15:01	DQB1*06:02	DQB1*06:02	DPB1*04:01	DPB1*240:01
G120	A*11	A*68	NA	NA	C*03	NA	DRB1*13	DRB1*15:01	DQB1*06:02	DQB1*06:04	DPB1*03:01	NA
G131	NA	NA	NA	NA	NA	NA	NA	NA	NA	NA	NA	NA
G140	NA	NA	NA	NA	NA	NA	NA	NA	NA	NA	NA	NA
G149	NA	NA	NA	NA	NA	NA	NA	NA	NA	NA	NA	NA
G154	A*03	A*02	B*35	B*42	C*04	C*07	DRB1*04	DRB1*15	DQB1*06:02	DQB1*03:02	NA	NA
G164	NA	NA	NA	NA	NA	NA	NA	NA	NA	NA	NA	NA
G165	A*03:01	A*02	B*15:01	B*44:03	C*03	C*16	DRB1*04:01	DRB1*15	DQB1*06:02	DQB1*03:02	NA	NA
G166	NA	NA	NA	NA	NA	NA	NA	NA	NA	NA	NA	NA
G189	A*02	A*01	B*57:01	NA	NA	NA	DRB1*04	DRB1*07:01	NA	NA	NA	NA
G208	A*24	A*32	B*40	B*44:02	C*03	C*07	DRB1*01:01	DRB1*04	DQB1*03:02	DQB1*05:01	DPB1*04:01	NA
G21	A*68:01	A*03:01	B*38	B*15	C*12	C*03	DRB1*04:01	DRB1*13:01	DQB1*06:03	DQB1*03:02	DPB1*131:01	NA
G24	A*01:01	A*24:02	B*57:01	B*39:06	C*07	C*06	DRB1*07:01	DRB1*08:01	DQB1*04	DQB1*03:03	NA	NA
G255	NA	NA	NA	NA	C*07	NA	NA	NA	NA	NA	NA	NA
G274	NA	NA	NA	NA	NA	NA	NA	NA	NA	NA	NA	NA
G28	A*68	NA	NA	NA	NA	NA	NA	NA	DQB1*06:02	NA	DPB1*04:01	NA
G289	NA	NA	NA	NA	NA	NA	NA	NA	NA	NA	NA	NA
G300	A*02:01	A*01:01	B*07:02	B*08:01	C*07	NA	DRB1*03:01	DRB1*15:01	DQB1*02:01	DQB1*06:02	DPB1*04:01	NA
G314	A*24:02	A*32:01	B*27:05	B*44	C*05	C*02	DRB1*04:01	DRB1*09:01	DQB1*03:02	DQB1*03:03	DPB1*04:01	DPB1*258:01
G33	A*26:01	A*68	B*51:01	B*07	C*15	C*07	DRB1*13:01	DRB1*15	DQB1*06:02	DQB1*06:03	NA	NA
G34	A*26:01	A*68:01	B*38	B*15	C*12	C*03	DRB1*13:01	DRB1*16:02	DQB1*06:03	DQB1*05:02	DPB1*54:01	DPB1*57:01
G348	NA	NA	NA	NA	C*07	C*04	DRB1*01	DRB1*15	DQB1*05:01	DQB1*06:02	DPB1*52:01	DPB1*442:01
G393	A*01:01	A*24:02	B*45:01	B*08:01	C*07:01	NA	DRB1*03:01	DRB1*04:01	DQB1*03:01	DQB1*02:01	DPB1*04:01	DPB1*20:01
G397	A*29	A*02	B*45	NA	NA	NA	DRB1*04:01	DRB1*10	DQB1*03:02	DQB1*05:01	NA	NA
G404	A*02	NA	B*08	B*15	C*03	C*07	NA	NA	DQB1*02:01	DQB1*03:02	NA	NA
G417	NA	NA	NA	NA	NA	NA	NA	NA	DQB1*06	NA	NA	NA
G427	NA	NA	B*15	NA	C*03	C*04	NA	NA	NA	NA	NA	NA

G43	A*02:01	A*11:01	B*51:01	B*07:02	C*07	C*15	DRB1*04:04	DRB1*15:01	DQB1*03:02	DQB1*06:02	DPB1*03:01	DPB1*46:01
G472	A*02	A*68	B*44	B*40:01	NA	NA	NA	NA	NA	NA	NA	NA
G48	A*24:02	A*02:01	B*39:06	B*57:01	NA	NA	DRB1*01:01	DRB1*07:01	DQB1*05:01	DQB1*03:03	DPB1*04:01	DPB1*04:02
G507	A*01	A*32	B*55	NA	C*03	C*04	DRB1*11	DRB1*13:02	DQB1*03:01	DQB1*06:04	NA	NA
G533	A*02	NA	B*15	NA	C*03	NA	DRB1*04	DRB1*15	DQB1*03:02	DQB1*06	NA	NA
G658	A*30	A*24	B*07	B*18	C*07	NA	NA	NA	DQB1*02	DQB1*06:02	DPB1*02:02	NA
G669	A*02:01	A*26:01	B*38	B*44:02	C05:01	C12:03	DRB1*15:01	DRB1*16:01	DQB1*06:02	DQB1*05:02	DPB1*04:01	NA
G708	A*02:01	NA	B*15:01	B*57:01	C*03	C*07:01	DRB1*04:01	DRB1*07	DQB1*03:02	DQB1*03:02	DPB1*04:01	DPB1*234:01
G712	A*02	A*03	B*07:02	B*08:01	C*07:01	C*07:01	DRB1*03:01	DRB1*15:01	DQB1*02:01	DQB1*06:02	DPB1*16:01	NA
G722	A*36	NA	NA	NA	NA	NA	DRB1*15	NA	DQB1*03	DQB1*06:02	NA	NA
G730	A*03:01	A*02	B*40:01	B*51:01	C*03	C*02	DRB1*01:01	DRB1*15:01	DQB1*05:01	DQB1*06:02	DPB1*04:01	NA
G738	A*02	A*03	NA	NA	NA	NA	NA	NA	DQB1*03	DQB1*06	NA	NA
G749	A*02	NA	NA	NA	C*03	NA	DRB1*15	NA	DQB1*06	NA	DPB1*169:02	NA
G750	NA	NA	B*35:03	B*56:01	C*01	C*04	DRB1*01	NA	DQB1*05:01	DQB1*06	NA	NA
G860	A*01:01	A*01:01	B*08:01	B*08:01	C*07:01	C*07:01	DRB1*03	DRB1*03	DQB1*02:01	DQB1*02:01	DPB1*04:01	DPB1*04:02
G864	A*02:01	A*26	B*08:01	B*40:01	C*03	C*07	DRB1*03:01	DRB1*13:02	DQB1*02:01	DQB1*06:04	DPB1*03:01	NA
G870	A*01	A*02	B*08	NA	NA	NA	NA	NA	DQB1*02:01	DQB1*06:02	NA	NA
G876	A*02:01	A*03:01	B*15:01	B*55:01	C*03	C*03	DRB1*04:01	DRB1*14:01	DQB1*03:02	DQB1*05:03	DPB1*90:01	NA
G896	A*01:01	A*02	NA	NA	C*07	NA	DRB1*03:01	DRB1*15:01	DQB1*02:01	DQB1*06:02	DPB1*138:01	NA
G911	A*02:01	NA	B*15	B*44:02	C*03	C*05	DRB1*04:01	DRB1*04:01	DQB1*03:01	DQB1*03	DPB1*04:01	NA
G912	A*26	A*03	B*40:01	B*44:03	C*03	C*16	DRB1*07	DRB1*09	DQB1*02:01	DQB1*03:03	DPB1*11:01	NA
G914	A*02:06	NA	NA	NA	NA	NA	NA	NA	DQB1*02	DQB1*05:01	DPB1*109:01	DPB1*136:01
G936	A*01:01	A*03:01	B*07:02	B*51:01	C*07	C*15	DRB1*04:04	DRB1*15:01	DQB1*03:02	DQB1*06:02	DPB1*04:01	NA
G939	A*03	A*26	B*15	NA	C*03	C*14	DRB1*01:01	DRB1*01:01	NA	NA	DPB1*04:01	NA
G942	A*11	NA	B*35	B*40:01	C*03	C*04	DRB1*04:03	DRB1*15:01	DQB1*03:10	DQB1*06:02	DPB1*16:01	DPB1*442:01
G943	NA	NA	NA	NA	NA	NA	NA	NA	NA	NA	NA	NA
G951	A*02:01	A*03:01	B*15	B*44:02	C*05	C*03:07	DRB1*04:01	DRB1*04:01	DQB1*03:01	DQB1*03:02	NA	NA
G973	A*02:01	A*31:01	B*40:01	B*44:02	C*03	C*05	DRB1*13:01	DRB1*13:01	DQB1*06:03	DQB1*06:04	DPB1*03:01	NA
G978	NA	NA	NA	NA	NA	NA	NA	NA	DQB1*02	DQB1*06	NA	NA

Table S8. TT / CC / TC alleles observed at SNP locus rs3135388 obtained with a PCR-based experiment together with allele calls at the HLA-DRB1 locus obtained with our approach for the 68 historical samples (data from Krause-Kyora et al. 2018).

Individual	rs3135388 genotype	DRB1 1	DRB1 2
G022	xxx	xxx	xxx
G102	CC	DRB1*04:03	DRB1*11
G104	CC	DRB1*04	xxx
G1042	xxx	xxx	xxx
G1044	CC	xxx	xxx
G1049	CT	DRB1*14:01	DRB1*15:01
G1065	CC	DRB1*01:01	DRB1*04:01
G1083	CC	DRB1*01	DRB1*08
G1137	CT	xxx	xxx
G1149	CT	DRB1*04:01	DRB1*15:01
G117	xxx	xxx	xxx
G118	CC	xxx	xxx
G119	TT	DRB1*15:01	DRB1*15:01
G120	CT	DRB1*13	DRB1*15:01
G131	xxx	xxx	xxx
G140	CT	xxx	xxx
G149	CC	xxx	xxx
G154	CT	DRB1*04	DRB1*15
G164	CT	xxx	xxx
G165	CT	DRB1*04:01	DRB1*15
G166	CC	xxx	xxx
G189	CC	DRB1*04	DRB1*07:01
G208	CC	DRB1*01:01	DRB1*04
G21	CC	DRB1*04:01	DRB1*13:01
G24	CC	DRB1*07:01	DRB1*08:01
G255	CT	xxx	xxx
G274	CC	xxx	xxx
G28	CT	xxx	xxx
G289	CT	xxx	xxx
G300	CT	DRB1*03:01	DRB1*15:01
G314	CC	DRB1*04:01	DRB1*09:01
G33	CT	DRB1*13:01	DRB1*15
G34	xxx	DRB1*13:01	DRB1*16:02
G348	CT	DRB1*01	DRB1*15
G393	CC	DRB1*03:01	DRB1*04:01
G397	CC	DRB1*04:01	DRB1*10
G404	CC	xxx	xxx
G417	CT	xxx	xxx
G427	CT	xxx	xxx
G43	CT	DRB1*04:04	DRB1*15:01
G472	CT	xxx	xxx
G48	CC	DRB1*01:01	DRB1*07:01
G507	CC	DRB1*11	DRB1*13:02
G533	CT	DRB1*04	DRB1*15
G658	CT	xxx	xxx
G669	CT	DRB1*15:01	DRB1*16:01
G708	CC	DRB1*04:01	DRB1*07
G712	CT	DRB1*03:01	DRB1*15:01
G722	CT	DRB1*15	xxx
G730	CT	DRB1*01:01	DRB1*15:01
G738	CT	xxx	xxx
G749	TT	DRB1*15	xxx
G750	CT	DRB1*01	xxx
G860	CC	DRB1*03	DRB1*03
G864	CC	DRB1*03:01	DRB1*13:02
G870	CT	xxx	xxx
G876	CC	DRB1*04:01	DRB1*14:01
G896	CT	DRB1*03:01	DRB1*15:01
G911	CC	DRB1*04:01	DRB1*04:01
G912	CC	DRB1*07	DRB1*09
G914	xxx	xxx	xxx
G936	CT	DRB1*04:04	DRB1*15:01
G939	CC	DRB1*01:01	DRB1*01:01
G942	CT	DRB1*04:03	DRB1*15:01
G943	xxx	xxx	xxx
G951	CC	DRB1*04:01	DRB1*04:01
G973	CC	DRB1*13:01	DRB1*13:01
G978	TT	xxx	xxx

Table S9. Validation of historical allele calls at class I HLA genes using OptiType.

	Optitype								TARGT pipeline						
Individual	A1	A2	B1	B2	C1	C2	Reads	Objective	A1	A2	B1	B2	C1	C2	HLA read depth
G102	A*02:01	A*68:01	B*18:01	B*44:02	C*03:03	C*07:01	20138	19231.77	A*02	NA	B*18	B*44	C*03	C*07	48.0
G1044	A*03:01	A*31:01	B*27:08	B*40:14	C*02:08	C*03:04	4658	4448.36	A*03:01	NA	NA	NA	NA	C*03	17.3
G120	A*11:01	A*68:01	B*07:33	B*40:01	C*03:04	C*07:02	32401	30942.935	A*11	A*68	NA	NA	C*03	NA	48.0
G154	A*02:01	A*03:01	B*07:02	B*35:02	C*04:01	C*07:02	10358	9985.082	A*02	A*03	B*42	B*35	C*04	C*07	41.3
G165	A*02:01	A*03:01	B*15:38	B*44:03	C*03:04	C*16:01	32745	31566.15	A*02	A*03:01	B*15:01	B*44:03	C*03	C*16	69.4
G189	A*01:01	A*02:01	B*40:01	B*57:01	C*03:04	C*06:02	3475	3318.615	A*01	A*02	NA	B*57:01	NA	NA	9.7
G24	A*01:01	A*24:02	B*39:06	B*57:01	C*06:03	C*07:02	59001	57407.963	A*01:01	A*24:02	B*39:06	B*57:01	C*06	C*07	174.3
G255	A*24:04	A*69:01	B*07:33	B*18:20	C*03:04	C*07:02	1084	1054.702	NA	NA	NA	NA	NA	C*07	3.7
G28	A*03:01	A*68:01	B*07:02	B*44:07	C*07:04	C*07:04	612	595.446	NA	A*68	NA	NA	NA	NA	1.6
G300	A*01:01	A*02:01	B*07:02	B*08:01	C*07:01	C*07:02	63023	60186.935	A*01:01	A*02:01	B*07:02	B*08:01	C*07	NA	241.3
G314	A*24:02	A*32:01	B*27:05	B*44:02	C*02:27	C*05:01	45719	44484.577	A*24:02	A*32:01	B*27:05	B*44	C*02	C*05	119.6
G33	A*26:01	A*68:01	B*07:02	B*51:01	C*07:02	C*15:02	28010	27001.62	A*26:01	A*68	B*07	B*51:01	C*07	C*15	69.0
G348	A*30:01	A*33:03	B*07:02	B*35:24	C*04:01	C*07:02	3520	3361.58	NA	NA	NA	NA	C*04	C*07	11.2
G393	A*01:01	A*24:02	B*08:01	B*45:04	C*06:02	C*07:01	45246	44024.338	A*01:01	A*24:02	B*08:01	B*45:01	NA	C*07:01	92.4
G397	A*01:23	A*02:01	B*45:01	B*57:01	C*07:17	C*12:03	2218	2138.112	A*29	A*02	B*45	NA	NA	NA	13.3
G404	A*02:13	A*26:01	B*08:01	B*15:38	C*03:04	C*07:02	1810	1728.52	A*02	NA	B*08	B*15	C*03	C*07	6.2
G427	A*02:01	A*03:07	B*15:05	B*35:14	C*03:04	C*04:01	1241	1196.294	NA	NA	B*15	NA	C*03	C*04	3.8
G472	A*02:01	A*68:01	B*40:01	B*44:02	C*03:67	C*05:01	1694	1617.74	A*02	A*68	B*40:01	B*44	NA	NA	5.0
G48	A*02:01	A*24:56	B*39:06	B*57:01	C*06:02	C*07:02	22650	21834.58	A*02:01	A*24:02	B*39:06	B*57:01	NA	NA	45.2
G533	A*02:01	A*03:01	B*15:39	B*40:01	C*03:04	C*03:04	1457	1430.754	A*02	NA	B*15	NA	C*03	NA	4.9
G658	A*24:02	A*30:02	B*07:02	B*18:01	C*05:01	C*07:02	1343	1294.652	A*24	A*30	B*07	B*18	NA	C*07	5.1
G708	A*02:01	A*02:01	B*15:38	B*57:10	C*03:04	C*07:01	24819	24372.238	A*02:01	NA	B*15:01	B*57:01	C*03	C*07:01	67.1
G712	A*02:01	A*03:01	B*07:02	B*08:01	C*07:01	C*07:02	43810	42232.81	A*02	A*03	B*07:02	B*08:01	C*07:01	C*07:01	155.9
G722	A*01:01	A*02:01	B*07:02	B*35:21	C*04:01	C*07:123	1104	1054.28	A*36	NA	NA	NA	NA	NA	3.0
G730	A*02:01	A*03:01	B*40:01	B*51:01	C*02:02	C*03:04	36987	35655.448	A*02	A*03:01	B*40:01	B*51:01	C*02	C*03	77.4
G738	A*02:01	A*03:22	B*07:02	B*15:01	C*03:04	C*07:02	3458	3333.462	A*02	A*03	NA	NA	NA	NA	9.6
G749	A*02:01	A*03:22	B*07:03	B*07:03	C*03:04	C*07:29	558	547.926	A*02	NA	NA	NA	C*03	NA	2.1
G750	A*03:01	A*24:02	B*35:33	B*56:01	C*01:02	C*04:01	4522	4359.188	NA	NA	B*35:03	B*56:01	C*01	C*04	17.9
G870	A*01:01	A*02:13	B*07:02	B*08:01	C*07:01	C*07:01	6460	6227.4	A*01	A*02	NA	B*08	NA	NA	14.8
G876	A*02:01	A*03:01	B*15:38	B*55:01	C*03:03	C*03:03	87099	83963.406	A*02:01	A*03:01	B*15:01	B*55:01	C*03	C*03	317.0
G896	A*01:01	A*02:01	B*07:02	B*08:01	C*07:01	C*07:01	6337	6108.848	A*01:01	A*02	NA	NA	C*07	NA	17.2
G911	A*02:01	A*03:01	B*15:07	B*44:02	C*03:04	C*05:01	6096	5931.388	A*02:01	NA	B*15	B*44:02	C*03	C*05	27.5
G912	A*03:01	A*26:01	B*40:01	B*44:03	C*03:04	C*16:01	6278	5938.968	A*03	A*26	B*40:01	B*44:03	C*03	C*16	17.7
G914	A*02:01	A*02:01	B*40:01	B*44:03	C*03:04	C*16:01	4447	4366.944	A*02:06	NA	NA	NA	NA	NA	7.0
G936	A*01:01	A*03:01	B*07:02	B*51:01	C*07:02	C*15:02	35611	34008.465	A*01:01	A*03:01	B*07:02	B*51:01	C*07	C*15	78.7
G939	A*03:01	A*26:01	B*15:01	B*51:13	C*03:03	C*14:04	5186	4952.57	A*03	A*26	B*15	NA	C*03	C*14	19.9
G942	A*11:01	A*33:03	B*35:24	B*40:01	C*03:04	C*04:01	3948	3770.32	A*11	NA	B*35	B*40:01	C*03	C*04	18.5
G951	A*02:01	A*03:01	B*15:01	B*44:02	C*03:04	C*05:01	7095	6839.57	A*02:01	A*03:01	B*15	B*44:02	C*03:07	C*05	18.1
G973	A*02:01	A*31:01	B*40:01	B*44:02	C*03:04	C*05:01	55211	52726.485	A*02:01	A*31:01	B*40:01	B*44:02	C*03	C*05	154.9

Note – Alleles reported in black are those with identical call from the two approaches. In blue are reported the cases for which we found read support for our allele call but not for the call by OptiType. Alleles reported in red are those for which we could not confirm the calls from our approach, but found supporting reads for the allele call by OptiType. In green are reported the allele calls we could not resolve. Called alleles that differed between the two approaches:

G154 - Optitype: B*07:02; TARGT: B*42. B*07:02 supported by 3 reads mapping to the second exon.
G165 - Optitype: B*15:38; TARGT: B*15:01. B*15:01 and B*15:38 differ in one position at the end of the second exon where the allele B*15:01 is supported by many reads while we did not find reads supporting B*15:38.
G393 - Optitype: B*45:04; TARGT: B*45:01. B*45:04 and B*45:01 differ in one position at the end of the second exon where B*45:01 is supported by many reads while we did not find reads supporting B*45:04.
G397 - Optitype: A*01:23; TARGT: A*29. Both the allele calls were supported by many reads and we could not resolve the allele calls.
G48 - Optitype: A*24:56; TARGT: A*24:02. A*24:02 supported by many reads while we did not find reads supporting A*24:56
G708 - Optitype: B*15:38-B*57:10; TARGT: B*15:01-B*57:01. B*15:01 supported by some reads while no reads supporting B*15:38. B*57:01 supported by some reads while no reads supporting B*57:10
G712 - Optitype: C*07:02; TARGT: C*07:01. C*07:02 supported by a small number of unique reads.
G722 - Optitype: A*01:01; TARGT: A*36. Both the allele calls were supported by many reads and was not and we could not resolve the allele calls.
G750 - Optitype: B*35:33; TARGT: B*35:03. Both the allele calls were supported by many reads and we could not resolve the allele calls.
G876 - Optitype: B*15:38; TARGT: B*15:01. B*15:01 supported by some reads while we did not find reads supporting B*15:38.
G914 - Optitype: A*02:01; TARGT: A*02:06. The two alleles differ in two position at the beginning of the first exon where A*02:01 supported by some reads but we did not find unique reads supporting A*02:06.
G951 - Optitype: C*03:04; TARGT: C*03:07. The allele C*03:04 is supported by more reads than C *03:07.

Table S10. HLA class I and class II allele frequencies at 1st field level for the 68 historical samples.

HLA-A			HLA-B			HLA-C			HLA-DRB1			HLA-DQB1			HLA-DPB1		
Allele	Frequency	n	Allele	Frequency	n	Allele	Frequency	n	Allele	Frequency	n	Allele	Frequency	n	Allele	Frequency	n
02	0.313	26	07	0.160	12	07	0.338	25	04	0.268	22	06	0.375	36	04	0.413	19
03	0.192	16	15	0.147	11	03	0.311	23	15	0.244	20	03	0.322	31	03	0.109	5
01	0.120	10	44	0.147	11	04	0.095	7	01	0.110	9	02	0.145	14	422	0.065	3
24	0.096	8	08	0.107	8	05	0.081	6	13	0.098	8	05	0.135	13	16	0.043	2
26	0.072	6	40	0.093	7	12	0.041	3	03	0.085	7	04	0.02	2	02	0.022	1
68	0.072	6	35	0.053	4	15	0.041	3	07	0.061	5				109	0.022	1
11	0.048	4	51	0.053	4	02	0.027	2	08	0.024	2				11	0.022	1
32	0.036	3	57	0.053	4	16	0.027	2	09	0.024	2				131	0.022	1
29	0.012	1	38	0.040	3	01	0.014	1	11	0.024	2				136	0.022	1
30	0.012	1	18	0.027	2	06	0.014	1	14	0.024	2				138	0.022	1
31	0.012	1	39	0.027	2	14	0.014	1	16	0.024	2				169	0.022	1
36	0.012	1	45	0.027	2				10	0.012	1				20	0.022	1
			55	0.027	2										234	0.022	1
			27	0.013	1										240	0.022	1
			42	0.013	1										258	0.022	1
			56	0.013	1										452	0.022	1
															46	0.022	1
															52	0.022	1
															54	0.022	1
															57	0.022	1
															90	0.022	1

Total number of 1st-field (2-digit) typed alleles at each locus: HLA-A = 83, HLA-B = 75, HLA-C = 74, HLA-DRB1 = 82, HLA-DQB1 = 96; HLA-DPB1 = 46.
Distinct 1st-field alleles at each locus: HLA-A = 12, HLA-B =16, HLA-C = 11, HLA-DRB1 = 12, HLA-DQB1 = 5; HLA-DPB1 = 21.

Table S11. HLA class I and class II allele frequencies at the 2nd field level for the 68 historical samples.

HLA-A			HLA-B			HLA-C			HLA-DRB1			HLA-DQB1			HLA-DPB1		
Allele	Frequency	n	Allele	Frequency	n	Allele	Frequency	n	Allele	Frequency	n	Allele	Frequency	n	Allele	Frequency	n
02:01	0.244	11	07:02	0.204	10	07:01	0.545	6	04:01	0.228	13	06:02	0.266	21	04:01	0.348	16
03:01	0.244	11	08:01	0.122	6	05:01	0.182	2	15:01	0.228	13	03:02	0.203	16	03:01	0.109	5
01:01	0.156	7	40:01	0.122	6	03:07	0.091	1	01:01	0.105	6	02:01	0.127	10	04:02	0.065	3
24:02	0.133	6	44:02	0.122	6	07:02	0.091	1	03:01	0.088	5	05:01	0.101	8	422:01	0.065	3
26:01	0.067	3	51:01	0.082	4	12:03	0.091	1	13:01	0.088	5	03:01	0.089	7	16:01	0.043	2
11:01	0.044	2	57:01	0.082	4				07:01	0.053	3	03:03	0.051	4	02:02	0.022	1
68:01	0.044	2	15:01	0.061	3				04:03	0.035	2	06:03	0.051	4	109:01	0.022	1
02:06	0.022	1	39:06	0.041	2				04:04	0.035	2	06:04	0.051	4	11:01	0.022	1
31:01	0.022	1	44:03	0.041	2				13:02	0.035	2	05:02	0.025	2	131:01	0.022	1
32:01	0.022	1	27:05	0.020	1				14:01	0.035	2	05:03	0.025	2	136:01	0.022	1
			35:01	0.020	1				08:01	0.018	1	03:10	0.013	1	138:01	0.022	1
			35:03	0.020	1				09:01	0.018	1				169:02	0.022	1
			45:01	0.020	1				16:01	0.018	1				20:01	0.022	1
			55:01	0.020	1				16:02	0.018	1				234:01	0.022	1
			56:01	0.020	1										240:01	0.022	1
															258:01	0.022	1
															452:01	0.022	1
															46:01	0.022	1
															52:01	0.022	1
															54:01	0.022	1
															57:01	0.022	1
															90:01	0.022	1

Total number of 2nd-field (4-digit) typed alleles at each locus: HLA-A = 45, HLA-B = 49, HLA-C = 11, HLA-DRB1 = 57, HLA-DQB1 = 79; HLA-DPB1 = 46.
Distinct 2nd-field alleles at each locus: HLA-A = 10, HLA-B =15, HLA-C = 5, HLA-DRB1 = 14, HLA-DQB1 = 11; HLA-DPB1 = 22.

Table S12. Pairwise global linkage disequilibrium estimates for the historical samples.

Locus pair	D'	Wn	p-value
A:B	0.804	0.844	0.0040*
A:C	1	1	0.0000*
A:DPB1	0.858	0.927	0.083
A:DQB1	0.764	0.709	0.0160*
A:DRB1	0.703	0.755	0.0430*
B:DPB1	0.829	0.823	0.0000*
B:DQB1	0.877	0.772	0.0000*
B:DRB1	0.882	0.775	0.0000*
C:B	0.833	0.829	0.0000*
C:DPB1	0	nan	0.0000*
C:DQB1	0.875	0.913	0.0000*
C:DRB1	0.833	0.882	0.0000*
DQB1:DPB1	0.85	0.882	0.459
DRB1:DPB1	0.929	0.917	0.186
DRB1:DQB1	0.984	0.883	0.0000*

Table S13. HLA two and three-locus haplotype frequencies (f > 0.03) for the historical samples calculated using the expectation-maximization algorithm.

Loci	Haplotype	Frequency	Copy number
A:B	A*0301:B*0702	0.19231	5
A:B	A*0101:B*0801	0.15385	4
A:B	A*0201:B*0702	0.07692	2
A:B	A*0201:B*4402	0.07692	2
A:B	A*2402:B*0702	0.07692	2
A:B	A*2402:B*3906	0.07692	2
A:C	A*0201:C*0501	0.33333	2
A:C	A*0101:C*0701	0.33333	2
A:DQB1	A*0201:0602	0.11765	4
A:DQB1	A*0101:0201	0.11765	4
A:DQB1	A*0301:0301	0.08824	3
A:DQB1	A*0301:0503	0.05882	2
A:DQB1	A*2402:0602	0.05882	2
A:DQB1	A*0301:0302	0.05882	2
A:DQB1	A*6801:0603	0.05882	2
A:DQB1	A*2402:0303	0.05882	2
A:DQB1	A*2601:0502	0.05882	2
A:DQB1	A*0201:0302	0.05882	2
A:DRB1	A*0301:0401	0.10969	3.7
A:DRB1	A*0201:1501	0.10969	3.7
A:DRB1	A*0301:1501	0.06678	2.3
A:DRB1	A*0201:0401	0.06678	2.3
A:DRB1	A*2402:1501	0.05882	2
A:DRB1	A*6801:1301	0.05882	2
A:DRB1	A*2402:0701	0.05882	2
A:DRB1	A*0101:0301	0.05882	2
A:DRB1	A*2402:0401	0.05882	2
A:DRB1	A*0301:1401	0.05882	2
B:DPB1	B*0801:DPB1*0401	0.16667	2
B:DPB1	B*5701:DPB1*0401	0.16667	2
B:DQB1	B*0702:DQB1*0602	0.22222	8
B:DQB1	B*0801:DQB1*0201	0.16667	6
B:DQB1	B*1501:DQB1*0302	0.08333	3
B:DQB1	B*5101:DQB1*0302	0.05556	2
B:DQB1	B*4001:DQB1*0604	0.05556	2
B:DRB1	B*0702:DRB1*1501	0.26667	8
B:DRB1	B*0801:DRB1*0301	0.13333	4
B:DRB1	B*3906:DRB1*0701	0.06667	2
B:DRB1	B*5101:DRB1*0404	0.06667	2
C:B	C*0701:B*0801	0.5	3
C:DQB1	C*0701:DQB1*0201	0.375	3
C:DQB1	C*0501:DQB1*0602	0.25	2
C:DRB1	C*0501:DRB1*1501	0.33333	2
DRB1:DQB1	DRB1*1501:0602	0.27273	12
DRB1:DQB1	DRB1*0401:0301	0.11364	5
DRB1:DQB1	DRB1*0301:0201	0.11364	5
DRB1:DQB1	DRB1*0401:0302	0.09091	4
DRB1:DQB1	DRB1*0101:0501	0.06818	3
DRB1:DQB1	DRB1*1301:0603	0.06818	3
DRB1:DQB1	DRB1*1401:0503	0.04545	2
DRB1:DQB1	DRB1*0404:0302	0.04545	2
A:B:C	A*0101:B*0801:C*0701	0.5	2
A:DRB1:DQB1	A*0201:DRB1*1501:DQB1*0602	0.125	4
A:DRB1:DQB1	A*0301:DRB1*0401:DQB1*0301	0.09375	3
A:DRB1:DQB1	A*0301:DRB1*1401:DQB1*0503	0.0625	2
A:DRB1:DQB1	A*2402:DRB1*1501:DQB1*0602	0.0625	2
A:DRB1:DQB1	A*6801:DRB1*1301:DQB1*0603	0.0625	2
A:DRB1:DQB1	A*0101:DRB1*0301:DQB1*0201	0.0625	2
B:DRB1:DQB1	B*0702:DRB1*1501:DQB1*0602	0.28571	8
B:DRB1:DQB1	B*0801:DRB1*0301:DQB1*0201	0.14286	4

B:DRB1:DQB1	B*5101:DRB1*0404:DQB1*0302	0.07143	2
C:DRB1:DQB1	C*0501:DRB1*1501:DQB1*0602	0.33333	2

Table S14. Allele calls at HLA –B and –DRB1 genes for 30 simulated aDNA samples.

Simulation ID	Read depth	B1	B2	DRB1 1	DRB1 2
genotype 1		B*07:02	B*40:08	DRB1*04:01	DRB1*15:01
simulation 1	1x	NA	NA	DRB1*04	NA
simulation 1	5x	B*07	B*40:08	DRB1*04	DRB1*15
simulation 1	10x	B*07	B*40:08	DRB1*04	DRB1*15
simulation 1	30x	B*07	B*40:08	DRB1*04:01	DRB1*15:01
simulation 1	60x	B*07:02	B*40:08	DRB1*04:01	DRB1*15:01
genotype 2		B*07:02	B*51:01	DRB1*10:04	DRB1*15:01
simulation 2	1x	NA	NA	NA	NA
simulation 2	5x	B*07	B*51	DRB1*10	DRB1*15
simulation 2	10x	B*07	NA	DRB1*10:04	DRB15:01
simulation 2	30x	B*07:02	B*51:01	DRB1*10:04	DRB15:01
simulation 2	60x	B*07:02	B*51:01	DRB1*10:04	DRB15:01
genotype 3		B*13:04	B*45:01	DRB1*01:01	DRB1*08:04
simulation 3	1x	NA	NA	DRB1*01	DRB1*08
simulation 3	5x	B*13:04	B*45:01	DRB1*01	NA
simulation 3	10x	B*13:04	B*45:01	DRB1*01:01	DRB1*08
simulation 3	30x	B*13:04	B*45:01	DRB1*01:01	DRB1*08:04
simulation 3	60x	B*13:04	B*45:01	DRB1*01:01	DRB1*08:04
genotype 4		B*51:01	B*13:04	DRB1*10:04	DRB1*01:01
simulation 4	1x	NA	NA	NA	NA
simulation 4	5x	B*51	B*13	DRB1*10:04	DRB1*01:01
simulation 4	10x	B*51:01	B*13	DRB1*10:04	DRB1*01:01
simulation 4	30x	B*51:01	B*13	DRB1*10:04	DRB1*01
simulation 4	60x	B*51:01	B*13:04	DRB1*10:04	DRB1*01:01
genotype 5		B*45:01	B*40:08	DRB1*08:04	DRB1*04:01
simulation 5	1x	NA	NA	NA	NA
simulation 5	5x	B*45:01	NA	NA	DRB1*04:01
simulation 5	10x	NA	NA	DRB1*08:04	NA
simulation 5	30x	NA	NA	DRB1*08:04	DRB1*04:01
simulation 5	60x	B*45:01	B*04:08	DRB1*08:04	DRB1*04:01
genotype 6		B*45:01	B*51:01 (153 T > C)	DRB1*08:04 (217 C > G)	DRB1*10:04 (164 G > T)
simulation 6	1x	NA	NA	NA	DRB*10
simulation 6	5x	NA	B*51 (153 T > C)	DRB1*08:04 (217 C > G)	DRB1*10:04 (164 G > T)
simulation 6	10x	B*45:01	B*51:01 (153 T > C)	DRB1*08:04 (217 C > G)	DRB1*10:04 (164 G > T)
simulation 6	30x	B*45:01	B*51:01 (153 T > C)	DRB1*08:04 (217 C > G)	DRB1*10:04 (164 G > T)
simulation 6	60x	B*45:01	B*51:01 (153 T > C)	DRB1*08:04 (217 C > G)	DRB1*10:04 (164 G > T)

Table S15. 31 individuals from the 1000 Genomes Project used in the validation test.

Individual	File1	File2	Population
NA19093	SRR100033_1	SRR100033_2	Yoruba (Africa)
NA19098	SRR077453_1	SRR077453_2	Yoruba (Africa)
NA19334	SRR100001_1	SRR100001_2	Luhya (Africa)
NA19332	SRR099997_1	SRR099997_2	Luhya (Africa)
HG00452	ERR031838_1	ERR031838_2	Han (East Asia)
HG00457	ERR031839_1	ERR031839_2	Han (East Asia)
NA20504	SRR748294_1	SRR748294_2	Toscani (Europe)
NA20505	SRR766033_1	SRR766033_2	Toscani (Europe)
HG00736	SRR099974_1	SRR099974_2	Puerto_Rico (Americas)
HG00737	SRR099984_1	SRR099984_2	Puerto_Rico (Americas)
HG01205	SRR098489_1	SRR098489_2	Puerto_Rico (Americas)
HG01241	SRR099990_1	SRR099990_2	Puerto_Rico (Americas)
HG01242	SRR098493_1	SRR098493_2	Puerto_Rico (Americas)
NA18501	SRR100022_1	SRR100022_2	Yoruba (Africa)
NA18504	SRR100028_1	SRR100028_2	Yoruba (Africa)
NA18516	SRR100026_1	SRR100026_2	Yoruba (Africa)
NA18517	ERR034551_1	ERR034551_2	Yoruba (Africa)
NA07000	SRR766039_1	SRR766039_2	Utah (European ancestry)
NA07037	ERR034542_1	ERR034542_2	Utah (European ancestry)
NA07048	SRR099452_1	SRR099452_2	Utah (European ancestry)
NA10851	SRR766044_1	SRR766044_2	Utah (European ancestry)
NA18939	SRR766031_1	SRR766031_2	Japan (East Asia)
NA18940	ERR034596_1	ERR034596_2	Japan (East Asia)
NA19794	SRR748785_1	SRR748785_2	Mexico (Americas)
NA19795	SRR708374_1	SRR708374_2	Mexico (Americas)
HG00097	SRR765989_1	SRR765989_2	Great_Britain (Europe)
HG00099	SRR765993_1	SRR765993_2	Great_Britain (Europe)
HG00100	SRR099966_1	SRR099966_2	Great_Britain (Europe)
NA18534	ERR034577_1	ERR034577_2	China (East Asia)
NA18536	ERR034578_1	ERR034578_2	China (East Asia)
NA18542	ERR031855_1	ERR031855_2	China (East Asia)

Table S16. SBT-based HLA-B genotypes for the 31 individuals from the 1000 Genomes Project used in the validation test. Information obtained from Gourraud et al. 2014.

Individual	B 1	B 2
NA19093	B*35:01:00	B*53:01:00
NA19098	B*51:01:00	B*53:01:00
NA19334	B*15:10	B*45:01/45:07
NA19332	B*15:10	B*58:02:00
HG00452	B*40:01:01/40:01:02/40:55	B*55:02:01
HG00457	B*39:01:01:01/39:01:01:02L/39:01:03/39:46	B*46:01:01/46:15N
NA20504	B*55:01:01/55:01:03	B*57:01:01
NA20505	B*35:03:01/35:70	B*57:01:01
HG00736	B*08:01:01/08:19N	B*58:01:01/58:11
HG00737	B*35:01:01/35:01:03/35:40N/35:42/35:57/35:94	B*51:01:01/51:01:05/51:01:07/51:11N/51:30/51:32/51:48/51:51
HG01205	B*07:02:01/07:02:06/07:02:09/07:44/07:49N/07:58/07:59/07:61	B*35:01:01/35:01:03/35:40N/35:42/35:57/35:94
HG01241	B*07:02:01/07:02:06/07:02:09/07:44/07:49N/07:58/07:59/07:61	B*15:03:01/15:103
HG01242	B*35:01:01/35:01:03/35:40N/35:42/35:57/35:94	B*44:03:01/44:03:03/44:03:04
NA18501	B*14:01	B*78:01:00
NA18504	B*15:03	B*39:10:00
NA18516	B*15:10	B*53:01:00
NA18517	B*49:01:00	B*51:01:00
NA07000	B*44:02:00	B*40:01:00
NA07037	B*15:10	B*40:01:00
NA07048	B*44:02:01:01	B*07:02
NA10851	B*40:01:00	B*08:01
NA18939	B*27:04:01	B*67:01:01
NA18940	B*46:01:00	B*52:01:00
NA19794	B*51:01:01/51:01:05/51:01:07/51:11N/51:30/51:32/51:48/51:51	B*56:01/56:24
NA19795	B*35:17:00	B*48:01:01/48:09
HG00097	B*07:02:01/07:02:06/07:02:09/07:44/07:49N/07:58/07:59/07:61	B*07:02:01/07:02:06/07:02:09/07:44/07:49N/07:58/07:59/07:61
HG00099	B*08:01:01/08:19N	B*44:02:01:01/44:02:01:02S/44:19N/44:27/44:66
HG00100	B*08:01:01/08:19N	B*57:01:01
NA18534	B*15:01:01:01/15:01:01:02N/15:01:06/15:01:07/15:102/15:104/15:140/15:146	B*58:01:01/58:11
NA18536	B*48:01:01/48:09	B*51:02:01
NA18542	B*46:01:00	B*58:01:00

Table S17. SBT-based HLA-DRB1 genotypes for the 31 individuals from the 1000 Genomes Project used in the validation test. Information obtained from Gourraud et al. 2014.

Individual	DRB1 1	DRB1 2
NA19093	DRB1*15:03	DRB1*13:01
NA19098	DRB1*01:02	DRB1*07:01
NA19334	DRB1*03:01:01:01/03:01:01:02	DRB1*08:04
NA19332	DRB1*03:01:01:01/03:01:01:02	DRB1*13:01:01
HG00452	DRB1*01:01:01	DRB1*11:01:01/11:01:08
HG00457	DRB1*15:01:01:01/15:01:01:02	DRB1*09:01:02
NA20504	DRB1*14:01:01/14:54	DRB1*04:04
NA20505	DRB1*16:01:01	DRB1*07:01:01:01/07:01:01:02
HG00736	DRB1*16:01:01	DRB1*03:01:01:01/03:01:01:02
HG00737	DRB1*13:01:01	DRB1*14:01:01/14:54
HG01205	DRB1*01:01:01	DRB1*13:01:01
HG01241	DRB1*15:01:01:01/15:01:01:02	DRB1*13:02:01
HG01242	DRB1*13:01:01	DRB1*13:01:01
NA18501	DRB1*01:02	DRB1*13:01
NA18504	DRB1*03:01	DRB1*01:02
NA18516	DRB1*08:04	DRB1*03:02
NA18517	DRB1*12:01	DRB1*13:03
NA07000	DRB1*03:01	DRB1*11:01:01/11:01:08
NA07037	DRB1*04:04	DRB1*13:02
NA07048	DRB1*04:01	DRB1*15:01
NA10851	DRB1*04:04:01	DRB1*07:01
NA18939	DRB1*15:01:01:01/15:01:01:02	DRB1*15:01:01:01/15:01:01:02
NA18940	DRB1*15:02	DRB1*08:02
NA19794	DRB1*01:01:01	DRB1*11:04:01
NA19795	DRB1*16:02:01	DRB1*08:02
HG00097	DRB1*15:01:01:01/15:01:01:02	DRB1*13:03:01
HG00099	DRB1*03:01:01:01/03:01:01:02	DRB1*11:01:01/11:01:08
HG00100	DRB1*03:01:01:01/03:01:01:02	DRB1*07:01:01:01/07:01:01:02
NA18534	DRB1*03:01:01:01/03:01:01:02	DRB1*04:06:01/04:06:02
NA18536	DRB1*15:01:01:01/15:01:01:02	DRB1*08:03:02
NA18542	DRB1*09:01	DRB1*03:01

Table S18. HLA genotypes obtained with our approach at HLA-B and HLA-DRB1 loci, for the 31 individuals from the 1000 Genomes Project used in the validation test.

Individual	B 1	B 2	DRB1 1	DRB1 2
NA19093	B*53:01:01G	B*53:01:01G	DRB1*13:02:01G	DRB1*15:03:01G
NA19098	B*51:01:01G	B*53:01:01G	DRB1*01:02:01G	DRB1*7:01:01G
NA19334	B*15:10:01	B*45:xx	DRB1*03:01:01G	DRB1*08:04:01
NA19332	B*15: xx	B*58:02:01	DRB1*03:01:01G/03:07:01G	DRB1*13:01:01G/13:27:01
HG00452	B*40:xx	B*55:02:01G	DRB1*01:01:01G	DRB1*11:01:01G
HG00457	B*39:01:01G	B*46:01:01G	DRB1*09:01:02G	DRB1*15:01:01G
NA20504	B*55:01:01G	B*57:01:01G	DRB1*04:04:01	DRB1*14:01:01G
NA20505	B*35:03:01G	B*57:01:01G	DRB1*07:01:01G	DRB1*16:01:01
HG00736	B*08:01:01G	B*58:01:xx	DRB1*03:01:01G	DRB1*16:01:01
HG00737	B*35:01:01G	B*51:01:01G	DRB1*13:01:01G	DRB1*14:01:01G
HG01205	B*07:02:01G	B*35:01:01G	DRB1*01:01:01G	DRB1*13:01:01G
HG01241	B*07:02:01G	B*15:03:01G	DRB1*13:02:01G	DRB1*15:01:01G
HG01242	B*35:01:01G/35:37:01	B*44:03:01G	DRB1*13:01:01G	DRB1*13:01:01G
NA18501	B*14:01:01G	B*78:01:01G	DRB1*01:02:01G	DRB1*13:01:01G
NA18504	B*15:03:01G	B*39:10:01	DRB1*01:23:01	DRB1*03:01:01G
NA18516	B*15:10:01	B*53:01:01G	DRB1*03:02:01	DRB1*08:04:01
NA18517	B*49:xx	B*51:01:01G	DRB1*12:01:01G	DRB1*13:03:01G
NA07000	B*40:01:01G	B*44:02:01G	DRB1*03:01:01G	DRB1*11:01:01G
NA07037	B*15:10:01	B*40:01:01G/40:30:01	DRB1*04:04:01	DRB1*13:02:01G
NA07048	B*07:02:01G	B*44:02:01G	DRB1*04:01:01G	DRB1*15:01:01G
NA10851	B*08:12:01	B*40:80:01	DRB1*04:04:01	DRB1*07:01:01G
NA18939	B*27:04:01G	B*67:01:01	DRB1*15:01:01G	DRB1*15:01:01G
NA18940	B*46:01:01G/46:32:01	B*52:01:01G	DRB1*08:02:01G	DRB1*15:02:01G
NA19794	B*51:01:01G	B*56:01:01G	DRB1*01:01:01	DRB1*11:04:01
NA19795	B*35:17:01	B*48:01:01G	DRB1*08:02:01G	DRB1*16:02:01G
HG00097	B*07:02:01G	B*07:02:01G	DRB1*13:03:01G	DRB1*15:01:01G
HG00099	B*08:01:01G	B*44:02:01G/44:20:01	DRB1*03:01:01G	DRB1*11:01:01G
HG00100	B*08:xx	B*57:01:01G	DRB1*03:01:01G	DRB1*07:01:01G
NA18534	B*15:xx	B*58:01:01G	DRB1*03:01:01G/03:05:01	DRB1*04:06:01G
NA18536	B*48:01:01G	B*51:02:01G	DRB1*08:03:02G	DRB1*15:01:01G
NA18542	B*46:01:01G	B*58:01:01G	DRB1*03:01:01G	DRB1*09:01:02G

Note – Alleles reported in black are those with identical call from the two approaches. In blue are reported the cases for which we found support for our allele call and we could not support the call provided in Gourraud et al. 2014. Alleles reported in red are instead those for which we could not confirm the calls from our approach. Called alleles that differed between the two approaches:

NA19093 - Gourraud et al. 2014: B*35:01:00; TARGT: B*53:01:01G, the former supported by a few reads.
NA19093 - Gourraud et al. 2014: DRB1*13:01; TARGT: DRB1*13:02:01G, the latter supported by six reads.
NA18504 - Gourraud et al. 2014: DRB1*01:02; TARGT: DRB1*01:23:01, the latter supported by 19 reads.
NA10851 - Gourraud et al. 2014: B*08:01; TARGT: B*08:12:01, the former supported by a few reads.
NA10851 - Gourraud et al. 2014: B*40:01:00; TARGT: B*40:80:01, the former supported by a few reads.
